# Bioactive Secondary Metabolites from Octocoral-Associated Microbes—New Chances for Blue Growth

**DOI:** 10.3390/md16120485

**Published:** 2018-12-04

**Authors:** Inês Raimundo, Sandra G. Silva, Rodrigo Costa, Tina Keller-Costa

**Affiliations:** Institute for Bioengineering and Biosciences (iBB), Instituto Superior Técnico (IST), Universidade de Lisboa, 1049-001 Lisbon, Portugal; nex_raimundo@hotmail.com (I.R.); sandragodinhosilva@tecnico.ulisboa.pt (S.G.S.)

**Keywords:** blue economy, biopharmaceuticals, bioprospection, host–microbe interactions, gorgonians, polyketides, terpenes

## Abstract

Octocorals (Cnidaria, Anthozoa Octocorallia) are magnificent repositories of natural products with fascinating and unusual chemical structures and bioactivities of interest to medicine and biotechnology. However, mechanistic understanding of the contribution of microbial symbionts to the chemical diversity of octocorals is yet to be achieved. This review inventories the natural products so-far described for octocoral-derived bacteria and fungi, uncovering a true chemical arsenal of terpenes, steroids, alkaloids, and polyketides with antibacterial, antifungal, antiviral, antifouling, anticancer, anti-inflammatory, and antimalarial activities of enormous potential for blue growth. Genome mining of 15 bacterial associates (spanning 12 genera) cultivated from *Eunicella* spp. resulted in the identification of 440 putative and classifiable secondary metabolite biosynthetic gene clusters (BGCs), encompassing varied terpene-, polyketide-, bacteriocin-, and nonribosomal peptide-synthase BGCs. This points towards a widespread yet uncharted capacity of octocoral-associated bacteria to synthetize a broad range of natural products. However, to extend our knowledge and foster the near-future laboratory production of bioactive compounds from (cultivatable and currently uncultivatable) octocoral symbionts, optimal blending between targeted metagenomics, DNA recombinant technologies, improved symbiont cultivation, functional genomics, and analytical chemistry are required. Such a multidisciplinary undertaking is key to achieving a sustainable response to the urgent industrial demand for novel drugs and enzyme varieties.

## 1. Introduction

**Figure 1:**
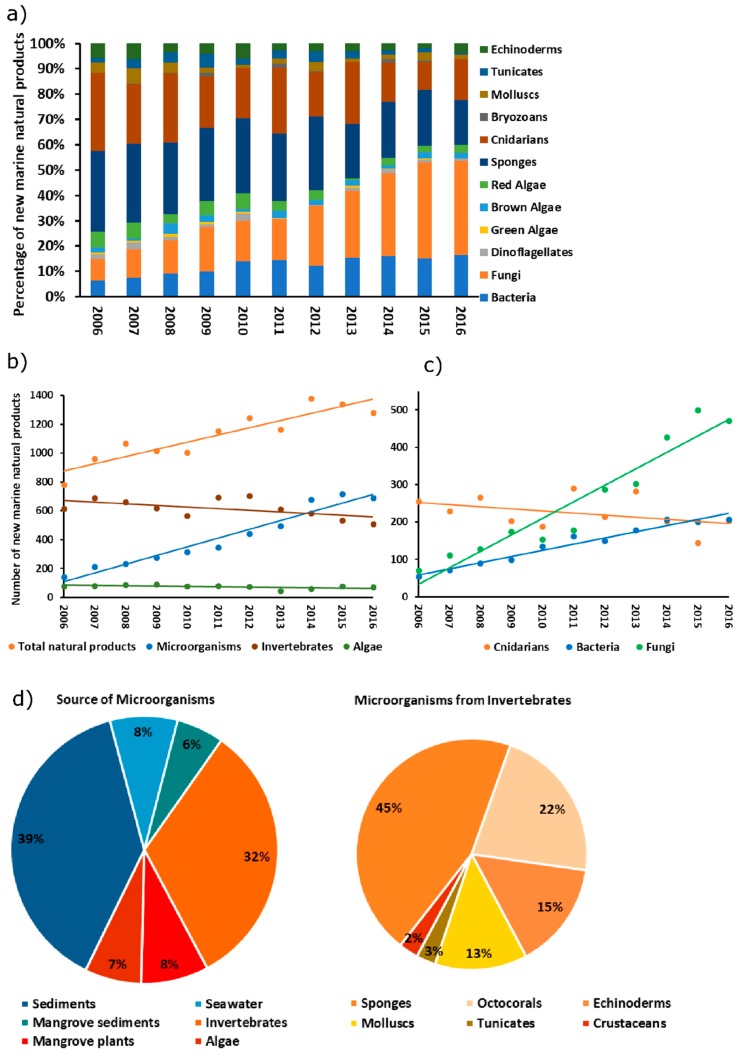
(**a**) Relative and (**b**,**c**) absolute numbers of new natural products discovered yearly from marine organisms over one decade (2006–2016). Data are based on inventories made by Blunt and colleagues in the 2008–2018 period [1,23,24,25,26,27,28,29,30,31,32]. The graphs clearly show an increase of natural products derived from marine microorganisms. Microorganisms (blue) in (**b**) include bacteria, fungi, and dinoflagellates; invertebrates (brown) include sponges, cnidarians, molluscs, bryozoans, ascidians and echinoderms; marine bacteria (blue) in (**c**) include cyanobacteria and *Mangrove bacteria*; marine fungi (green) include Mangrove fungi. (**d**) Habitat (source) of marine microorganisms (bacteria, fungi, and dinoflagellates) from which novel natural products have been discovered during 2013 (data based on reference [24], sediment and marine invertebrate animals being the dominating habitats). The pie chart on the right-hand side provides a deeper insight into the diversity of the invertebrates that host the producing symbionts, revealing sponges and octocorals as dominating animals.

Marine organisms produce a large diversity of unusual, often highly complex, natural products. In fact, the number of new chemical structures reported from marine biomes has been continuously increasing over the past decade (Figure 1a). With over 200 novel chemical structures described every year [1], corals (Cnidaria, Anthozoa) are the second most prolific source of natural products retrieved from marine animals after sponges. Since the first chemical studies on Caribbean gorgonian corals of the genus *Pseudopterogorgia* in the 1980s [2], corals in the subclass Octocorallia have been considered promising reservoirs of bioactive natural products, including a wealth of novel, unusual terpenoids [3,4,5,6]. For example, the diterpenoids caribenols A and B, and elisapterosin B isolated from *Pseudopterogorgia elisabethae* (now *Antillogorgia elisabethae*), along with bipinnapterolide B from *P. bipinnata*, are promising antituberculosis compounds, inhibiting *Mycobacterium tuberculosis* growth in vitro [3,7]. Pseudopterosin A, a diterpene with anti-inflammatory activity, also obtained from *P. elisabethae* and its dinoflagellate (*Symbiodinium* sp.) symbiont, has already undergone Phase II human clinical trials where it showed increased reepithelization and accelerated wound-healing [7]. Octocorals lack the physical protection of a massive calcium carbonate skeleton typical for scleractinian corals, and instead rely heavily on chemical defence mechanisms to resist predation and avoid overgrowth and fouling [8]. Their chemical arsenal encompasses not only a variety of terpenoids, particularly sesqui- and diterpenoids, but also steroids, alkaloids, and polyketides [1,7,9,10]. Many of these natural products possess potent antibacterial, anti-inflammatory, anticancer, antiviral, antimalarial, and neuroprotective properties [7,10] and are thus of enormous potential for the blue economy sector. Nevertheless, commercial development of coral-derived drugs is scarce, impaired by the fact that large-scale chemical synthesis of complex metabolites is usually unprofitable, and the harvest of large amounts of wild organisms highly unsustainable. Hence, very few compounds complete clinical trials and find their way into the market. Since coral-derived drug reports have traditionally focused on tissue extracts, commonly ignoring the biosynthetic origin of the compounds in question, it is often unclear whether the actual producer is the animal itself or the associated microbial community. Research on marine sponges and bryozoans, for instance, has shown that their bioactive compounds often possess a symbiont origin, revealing marine invertebrates as true hotspots of microbial metabolic versatility [11,12]. We now know that polyketide and terpene synthases are indeed widespread in microorganisms [13,14,15,16], and several polyketides and terpenoids have already been identified in marine microbes [17,18,19]. Current natural-product discovery from marine microorganisms follows an ever-increasing trend, surpassing the numbers of novel invertebrate-derived compounds described per year (Figure 1a,b). In 2006, less than 20% of all newly discovered marine natural products derived from microbes (including fungi, bacteria, and dinoflagellates), whereas nearly 80% were registered for invertebrates. In contrast, in 2016 microorganisms were the source of over 50% of all novel marine natural products, while 40% still came from invertebrates (Figure 1a). The absolute number of new natural products from marine microorganisms has increased five times over the past decade (Figure 1b), and continued effort to captivate marine microbial life in the laboratory is suggested as a pivotal contribution to this trend. Nearly half (47%) of the marine microbes from which novel compounds were retrieved in 2013, for example, had been isolated from host organisms, being marine invertebrates, particularly sponges and octocorals, the most popular hosts (Figure 1c,d). Therefore, new technologies that focus on symbiont-driven production of coral-derived bioactive molecules have the potential to overcome current hindrances in the commercialization of these drugs, contributing to a more sustainable blue economy. Although previous studies have addressed the structural complexity and remarkable biotechnological potential of coral-derived natural products [5,7,20,21], the role of octocoral-associated microbes in the production of these compounds has received little attention. Because of the steep advances in current octocoral microbiology research [22] and of the discovery, in recent years, of highly bioactive natural products from octocoral-associated microbes (see sections below), here we interrogate the microbiomes of these animals as a natural reservoir of novel molecules with inhibitory properties. This review therefore provides a comprehensive inventory of over 80 promising bioactive compounds (Table S1) and various enzymes (Table S2) derived from microbes associated with octocorals. It delineates the major octocoral-associated microbial groups involved in the biosynthesis of natural products, focusing on the astonishing chemical diversity and bioactivity spectrum of these compounds while approaching their medical, pharmaceutical and biotechnological potential. We further employ functional genomics to valorize this potential in an unprecedented fashion, revealing 440 predicted secondary metabolite biosynthetic gene clusters (BGCs) across the genomes of 15 bacterial associates of octocorals, 11 of which corresponding to original genome submissions. We finally debate on future research directions and methodologies leading to minimally invasive and economically reliable retrieval of bioactive secondary metabolites from the highly diverse and chemically complex microbial communities that inhabit octocorals.

## 2. Diversity and Function of the Octocoral Microbiome

Corals are benthic, sessile, suspension-feeding marine invertebrates that live in symbiosis with complex microbial communities, comprised of endosymbiotic dinoflagellates and endolithic algae, fungi, alveolates, bacteria, archaea, and viruses [22,33]. This consortium, consisting of the animal and its associated community of internal and external microorganisms, is commonly referred to as the holobiont [34]. The associated microbes deliver an extra supply of carbon and nitrogen to their host, participate in nutrient cycling and detoxification, UV protection, genetic exchange, and chemical defence of the animal [22,34,35]. Corals are divided into two subclasses, the Hexacorallia, which comprise sea anemones and reef-building hard corals (order Scleractinia) and the Octocorallia, which comprise the orders Helioporacea (commonly referred to as “blue corals”), Pennatulacea (so-called “sea pens”), and Alcyonaceae (including organisms popularly named as “sea fans”, “sea whips”, and “gorgonians”) [36]. Organisms in the latter two orders are also commonly referred to as “soft corals”. The term “gorgonian coral”, in its turn, is commonly applied to multiple species in the Alcyonaceae order, although the formally accepted taxon Gorgoniidae corresponds to only one of the several families within this order. Octocorals, characterized by the eightfold symmetry of their polyps, are ubiquitous in the world’s oceans, and occur from arctic to tropical waters and from shallow reefs to the deep sea. This highly diverse taxon consists of more than 3000 extant species in at least 47 validated families [36]. As true ecosystem engineers, octocorals structure benthic ecosystems where they may contribute to up to 95% of the total biomass [37,38], determine carbon flux [39], and substantially increase benthic biodiversity [38,40]. Octocoral–microbe interactions have been reviewed recently by van de Water and colleagues [22]. Octocorals in the euphotic zone live in mutualistic association with photosynthetic dinoflagellates of the genus *Symbiodinium*, which supplies the animal with a substantial amount of energy in form of carbohydrates and participates in nitrogen and phosphorus cycling [22]. *Symbiodinium* and other microalgal symbionts produce the organic sulphur compound dimethylsulfoniopropionate (DMSP) that can be detected in high concentrations in the tissue of their coral hosts. Coral-associated bacteria of the *Roseobacter* clade (*Alphabroteobacteria*) and certain *Gammaproteobacteria* of the orders *Oceanospirillales*, *Alteromonadales* and *Vibrionales* can breakdown DMSP, playing important roles in sulphur cycling in the coral holobiont [41]. Yet the numerous octocorals that colonize deeper waters lack *Symbiodinium* symbionts and primarily rely on chemotrophic feeding and interactions with prokaryotic symbiont communities [22,42,43]. 

Octocoral–bacterial communities are distinct from the surrounding seawater and can be quite diverse in some host species [44] or dominated by a few core microbial taxa in others [22,45,46]. *Proteobacteria* are usually the most represented phylum, followed by *Actinobacteria* and *Bacteroidetes*. A core consortium of *Oceanospirillales* phylotypes related to the genus *Endozoicomonas* is frequently reported in octocorals and can make up to over 96% of the bacterial community [22]. Other commonly detected orders are *Rhodobacterales*, *Vibrionales*, *Alteromonadales*, and *Cellvibrionales*, yet bacterial community composition may vary substantially between host species [22]. For example, the highly valuable, ornamental octocoral *Corallium rubrum* is highly dominated by *Spirochaetales*, while *Endozoicomonas*-like phylotypes are in the minority [47]. Genomic and phenotypic studies suggest that coral/octocoral-associated bacteria may occupy many functional niches within the holobiont, playing roles in nutrient exchange and cycling, amino acid synthesis, and host chemical defence through antibiotic production, thereby actively contributing to shaping the structure of the microbiomes they live in [22,35].

## 3. Octocoral-Associated Microbes as Natural-Product Manufacturers

**Figure 2:**
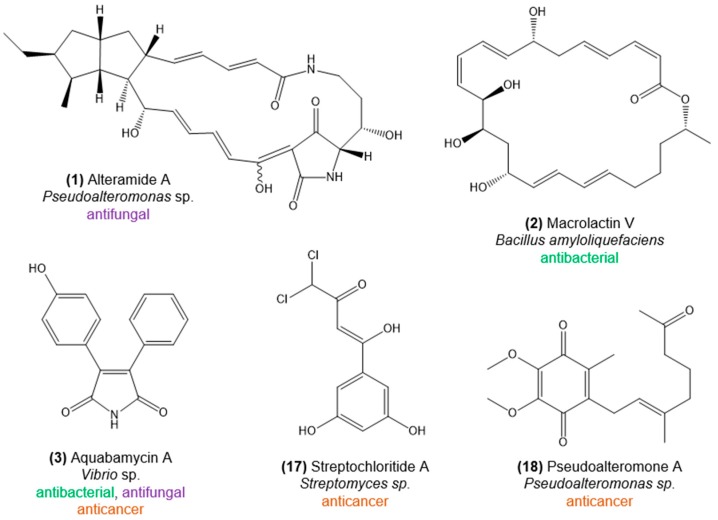
Chemical structures of bioactive natural products from bacteria associated with octocorals. The compounds exhibit antibacterial, antifungal, anticancer, or anti-inflammatory activity. Further information is provided in Table S1.

It has long been suspected that natural-product biosynthesis by microbial symbionts could contribute significantly to the chemical diversity commonly reported for sessile marine invertebrates [11,15]. In the past 20 years, the status of marine symbiotic bacteria as true producers of several chemical structures underlying manifold bioactivities was solidified. Thanks to the application of metagenomics-based strategies, much evidence emerged for a prokaryotic genome architecture behind the biosynthesis of highly potent compounds often inferred to be produced by so-far uncultivatable symbionts [11,12,16,48]. The cultivation-independent discovery of the bryostatin, onnamide, theopederin, psymberin, and ET743 biosynthetic gene clusters, to name a few, and their respective assignment to the genomes of bacterial symbionts (see reference [49] for a review), has considerably furthered our understanding of the ecology and evolution of important marine invertebrate models in natural products research such as marine sponges, bryozoans, tunicates, and scleractinian corals. In contrast, dedicated metagenomics-based assessments of natural-product biosynthesis capacities within octocoral microbiomes have yet to be performed, despite increasing knowledge of their diversity enabled by cultivation-independent approaches [22,44]. Octocoral-associated bacteria presently known to produce bioactive compounds belong to the genera *Streptomyces* (*Actinobacteria*), *Bacillus* (*Firmicutes*), *Vibrio*, and *Pseudoalteromonas* (both *Gammaproteobacteria*), all obtained in culture. Despite the limited diversity of producers documented to date, the synthesized natural products encompass alkaloids, maleimides, polyketides, and terpenoids with antibacterial, antifungal, antiprotozoal, and cytotoxic activities (Figure 2, Table S1).

**Figure 3:**
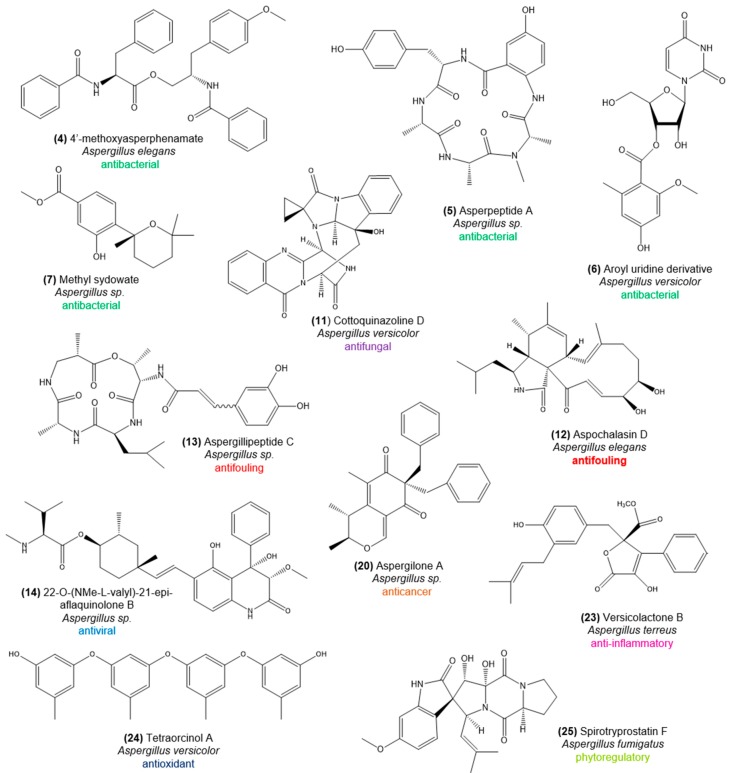
Chemical structures of bioactive natural products from octocoral-associated *Aspergillus* spp. The activity spectrum of the presented compounds comprises antiviral, antibacterial, antifouling, anticancer, anti-inflammatory, antioxidant, and phytoregulatory effects. Further information is provided in Table S1.

**Figure 4:**
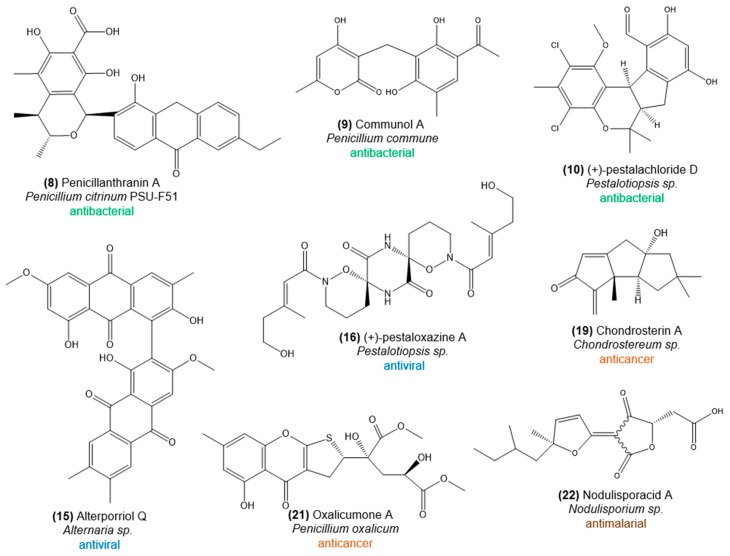
Chemical structures of bioactive natural products from fungi associated with octocorals (other than *Aspergillus* spp.). The compounds exhibit antiviral, antibacterial, anticancer, or antimalarial activity. More information is provided in Table S1.

Knowledge of fungal diversity in octocorals is primarily based on cultivation-dependent strategies and hence restricted to taxa more amenable to cultivation. Nevertheless, those cultured representatives have been a splendid source of bioactive natural products, often with unusual chemical structures (Figure 3 and Figure 4), making octocoral-associated fungi a wealthy target for bioprospecting. Ascomycota is the most frequently cultured phylum from octocorals, with Eurotiomycetes as the most prominent class and *Aspergillus* and *Penicillium* as dominating genera [22,50,51]. A variety of Sordariomycetes and Dothidiomycetes fungi are also commonly isolated from octocorals around the world, including the genera *Nigrospora*, *Fusarium*, and *Cladosporium* [22,50,51]. Little is known about the function of these fungi in the coral host, and especially the role of the most frequently isolated genus *Aspergillus* is controversial. While *Aspergillus sydowii* has been repeatedly associated with fungal disease (aspergillosis) in several host species (e.g., reference [52]), beneficial roles have been suggested for octocoral-associated fungi because of the diverse and highly active natural products they produce, displaying strong antibacterial or antifouling activities (e.g., reference [51]). Currently, *Aspergillus* spp. are clearly the most prolific producers of natural products among octocoral-associated microbes, as more than 35 bioactive compounds have been described from these species in the last decade. Other bioactive ascomycetes from octocorals are *Alternaria*, *Cochliobolus*, *Nodulisporium*, *Penicillium*, *Pestalotiopsis*, *Phoma*, *Trichoderma*, and *Xylariaceae* (Table S1). The diversity of the natural products they synthesize and their activity spectrum is enormous, encompassing anthraquinones, aromatic butenolids, chromones, cyclopeptides, indole alkaloids, lactones, macrolides, orcinols, phenylalanines, polyketides and terpenoids with antiviral, antioxidant, antibacterial, antifungal, antifouling, antimalarial, and anticancer activity (Table S1). 

Below, we provide a comprehensive overview of bioactivities registered for, primarily, bacteria and fungi associated with octocorals. While many of the covered compounds are highlighted in Figure 2, Figure 3 and Figure 4, a full list of all bioactive compounds documented in this article, along with details on host and microbe identities, is provided in Table S1.

### 3.1. Antibacterial and Antifungal Activity

To cure and prevent infections in humans and animal husbandry, an estimated amount of 100,000 tons of antibiotics is manufactured annually worldwide, resulting in the selection of pathogenic bacteria resistant to multiple drugs [53]. An infamous case is that of methicillin-resistant *Staphylococcus aureus* (MRSA), which is resistant not only to methicillin but usually also to aminoglycosides, macrolides, tetracycline, chloramphenicol, and lincosamides, acting as a major source of hospital-acquired infections [54,55]. Fungal infections are likewise a serious problem as they cause critical yield losses in the agricultural sector, as well as unpleasant (and eventually severe) diseases, such as candidiasis, in humans. Thus, there is a continued and increasing demand for novel antibiotics and fungicides from the health and agricultural sectors. Fungi and bacteria associated to octocorals have been reported to produce antimicrobial compounds showing activity against Gram-negative and Gram-positive bacteria, filamentous fungi, and yeasts (Table S1).

*Pseudoalteromonas* sp. strain OT59, isolated from a healthy *Leptogorgia alba* specimen, produces the polyketide ateramide A (**1**), a compound that displays light-dependent antifungal activity [56]. In the darkness, both strain OT59 and pure alteramide A inhibit the growth of the ascomycete fungus *Penicillium citrinum* isolated from the necrotic tissue of a *Eunicea* sp. specimen. In fact, alteramide A also inhibits several other *Penicillium*, *Aspergillus*, *Fusarium*, and *Trichoderma* species. Strain OT59 produces larger quantities of alteramide A in the dark, while light exposure inactivates the compound through photo-induced cyclization. These findings imply that coral-associated bacteria could protect their host from infections during heterotrophic night feeding, when coral polyps are more exposed. However, alteramide A (isolated previously also from a sponge-derived *Alteromonas* sp. [57]) induces changes in fungal metabolite distribution, promoting higher production of the bacteriostatic antibiotic citrinin and of the mycotoxin citrinadin, which suggests a defensive response of the fungus and highlights the complexity of multipartite interactions. 

*Bacillus amyloliquefaciens* strain SCSIO 00856, isolated from the octocoral *Junceella juncea*, produces the macrolide macrolactin V (**2**), which is strongly active against *Escherichia coli*, *Bacillus subtilis*, and *Staphylococcus aureus*, with a minimum inhibitory concentration (MIC) value of 0.1 μg mL^−1^ [58]. Malemeid derivatives aquabamycin A–G (**3**), active against *Micrococcus luteus*, *Bacillus subtilis*, *Proteus vulgaris,* and *Escherichia coli* and the fungus *Nematospora coryli*, are produced by the *Vibrio* sp. strain WMBA, isolated from the surface of *Sinularia polydactyla* [59]. In a dual-culture overlay plate assay, two *Alphaproteobacteria* isolates, strain SC4TGZ4 and *Pseudovibrio* sp. SC4TGZ3, retrieved from *Sinularia* sp., showed strong activity against multidrug resistant (MDR) *Mycobacterium tuberculosis* clinical strains, causative agents of tuberculosis (TB) disease [60]. A similar bioassay was used by Radjasa and Sabdono [61] to assess the antibacterial activity of *Sinularia*-associated bacterial isolates, whereby actinobacterium strain *Athrobacter* sp. SFNB.5 was found to inhibit the growth of *Vibrio*, *Staphylococcus*, and *Tenacibaculum* species. Nonribosomal peptide synthase (NRPS) encoding genes were amplified from the genomic DNA of *Athrobacter* sp. SFNB.5 and suggested to be involved in the observed antibacterial activity. The authors also discuss the potential ecological role of *Athrobacter* in host defence through the inhibition of biofilm formation by bacterial pathogens. 

*Aspergillus elegans* strain ZJ-2008010, isolated from *Sarcophyton* sp. (Alcyoniidae), produces the phenylalanine derivatives 4′-*O*-methoxyasperphenamate (**4**) and asperphenamate. These compounds have shown activity against *Staphylococcus epidermidis* [62], a common member of the human skin microbiome but also a causative agent of chronical infections in patients carrying implanted medical devices, including prosthetic joints, heart valves, and pacemakers [63]. Other agents active against *S. epidermidis* are the cyclopeptide asperpeptide A (**5**) and derivatives of the nucleoside aroyl uridine (**6**) produced by *Aspergillus* sp. XS-20090B15 (from the host *Muricella abnormalis*) and *Aspergillus versicolor* (from the host *Dichotella gemmacea*), respectively [64,65]. Another *Aspergillus* sp. strain (ZJ-2008001) from *D. gemmacea* is the source of phenolic bisabolane-type sesquiterpenoids, including methyl sydowate (**7**) which is moderately active against *S. aureus* [66]. These compounds were retrieved from marine sponges and the octocorals *Pseudopterogorgia rigida*, *Muricia elongata* and *Plexaurella nutans* [66] as well, indicating that microbial associates may produce such compounds within their hosts in significant amounts. The anthraquinone penicillanthranin A (**8**), obtained from *Penicillium citrinum* PSU-F51 associated with the sea fan *Annella* sp., is active against MRSA strains with an MIC value of 16 µg/mL [67]. *Penicillium commune* strain 518, isolated from *Muricella abnormalis*, is the source of several aromatic polyketides, including communol A (**9**), F, and G, moderately active against *E. coli* and *Enterobacter aerogenes* [68]. *Pestalotiopsis* sp. ZJ-2009-7-6, isolated from *Sarcophyton* sp., is the producer of the benzophenone derivative ()-pestalachloride D (**10**), which is active against several Gram-negative bacteria [69]. *Aspergillus versicolor* strain LCJ-5-4 from the octocoral *Cladiella* sp., a rich source of cyclopentapeptides and radical-scavenging polyketides [70], also produces the antifungal alkaloid cottoquinazoline D (**11**) that suppresses the growth of *Candida albicans* in vitro with an MIC value of 22.6 μM [71]. 

### 3.2. Antifouling Activity

Biofouling is characterized by the superposition of organisms on surfaces, starting with bacterial biofilm formation and advancing with the settlement of micro- and macroalgae and invertebrate larvae [72]. It is a common problem of underwater structures all over the world and an economic threat for shipping, offshore aquaculture, and coastal industries. To prevent this problem, tributyltin (TBT) compounds and other highly ecotoxic chemicals have been used in antifouling paints on hulls of ocean-faring vessels. However, due to the enormous environmental threat that TBT compounds present to marine organisms, their application has been completely banned by the International Maritime Organization (IMO) in 2008 [73]. With the ban of TBT-containing antifoulants, research interest in natural products with antifouling activities has been continuously growing. Marine sessile invertebrates, including sponges and corals, and their associated microbes constitute a promising and environmentally friendly source of secondary metabolites that inhibit the settlement and growth of biofouling-causing organisms [72,74].

Octocoral-derived terpenoids are well known for their antifouling properties. For example, the gorgonian coral *Junceella juncea* is the source of briaran-type diterpenoids juncins R-ZI, juncin ZII, gemmacolide A and B, and junceellolide D, all showing potent activity against barnacle (*Balanus amphitrite*) larvae settlement with EC50 values from 0.004 to 21.06 μg/mL [75]. There is, in fact, a growing number of reports on antifouling compounds produced by marine microbial symbionts, reviewed recently by Satheesh et al. [72]. Dobretsov and Qian [76] assessed the antifouling effect of epibiotic bacteria isolated from the surface of the octocoral *Dendronephthya* sp. Two strains, namely, a *Vibrio* sp. and an unidentified alphaproteobacterium, were found to reduce settlement of the tubeworm *Hydroides elegans* larvae below 10% compared to over 80% larval settlement in controls. Cochliomycin A, produced by the fungus *Cochliobolus lunatus* isolated from the gorgonian *Dichotella gemmaceae*, shows strong antifouling activity against larval settlement of the barnacle *B. amphitrite* with an EC50 value of 1.2 µg/mL (Table S1, [77]). The same gorgonian species is the host of *Penicillium* sp. producing the polyketide 6,8,50,60-tetrahydroxy-30-methylflavone, which shows significant antifouling activity against *B. amphitrite* larvae settlement with an EC50 value of 6.7 µg/mL (Table S1, [78]). *Aspergillus elegans* strain ZJ-2008010, source of antibacterial phenylalanine derivatives (see previous section), also produces aspochalasins D (**12**), I, J, and H, which are cytochalasins that display strong antifouling activity against *B. amphitrite* larvae [62]. Aspergillipeptide C (**13**), a cyclic tetrapeptide obtained from *Aspergillus* sp. SCSGAF 0076 associated with the octocoral *Melitodes squamata,* strongly inhibits larvae settlement of the bryozoan *Bugula neritina* with an EC50 value of 11 µg/mL [79]. Similar antifouling activities against *B. neritina* larvae were reported for indole alkaloids Cyclotryprostatin B and fumiquinazoline D, produced by *Aspergillus sydowii,* associated with the octocoral *Verrucella umbraculum* (Table S1, [80]). Further antifouling compounds and their source are given in Table S1. Overall, our review uncovered a multitude of compounds from octocoral-associated microbes showing EC50 values much lower than the standard requirement (EC50 = 25 µg/mL) established by the U.S. Navy program as an efficacy level for natural antifouling. Testimony to this emerging potential is the fact that some antifouling coatings based on natural products from marine organisms have already found their way into the market, e.g., under the trade names PearlSafe^®^TM, NetSafe^®^TM and SEA-NINE™ 211N [72]. SEA-NINE™ 211N, a 30% solution of 4,5-dichloro-2-n-octyl-4-isothiazolin-3-one (DCOIT), is a rapidly biodegradable antifouling agent developed by the Rohm and Haas Company^®^, Philadelphia, PA, USA (now Dow Chemical Company^®^) for a new generation of more environmentally acceptable antifouling paints for ships and underwater structures.

### 3.3. Antiviral Activity

Viruses are often highly contagious, causing a variety of severe or even fatal infections in humans and livestock, frequently at epidemic or pandemic scales. Available treatments for viral infections are limited whereas resistance of viruses to the treatments in place is increasing, many times leaving the alleviation of symptoms with analgesics as the only option. Therefore, the search for novel antiviral drugs that allow an effective control of viral infections is paramount. Natural products with antiviral activity retrieved from marine organisms have been reviewed by Cheung et al. [81] and Moghadamtousi et al. [82]. A variety of marine fungi, including the genera *Aspergillus*, *Penicillium*, *Fusarium*, and *Cladosporium*, produce secondary metabolites with strong antiviral activity, holding promise for further drug development [82]. 

From an ethyl acetate extract prepared from *Aspergillus* sp. XS-20090B15, isolated from the octocoral *Muricella abnormalis*, the hydroquinolone 22-*O*-(NMe-l-valyl)-21-epi-aflaquinolone B (**14**) was obtained. It showed strong activity (IC50 = 0.042 µM) against the respiratory syncytial virus (RSV), a common cause of respiratory tract infections in humans that can also lead to the development of bronchiolitis and pneumonia [83]. Alkaloids 9a,14-dihydroxy-6b-*p*-nitrobenzoylcinnamolide, and 7a,14-dihydroxy-6b-*p*-nitrobenzoylconfertifolin from *Aspergillus* sp. strain SCSGAF 0076, isolated from the octocoral *Melitodes squamata*, and the cyclopeptide asperterrestide A produced by *Aspergillus terreus* strain SCSGAF0162, isolated from *Echinogorgia aurantiaca*, inhibit the common human influenza virus A subtypes H1N1 and H3N2 (Table S1, [84,85]). Strain SCSGAF0162 further produces the butyrolactone derivative isobutyrolactone II and the territrem derivative 11a-dehydroxyisoterreulactone A, which possess activity towards the ubiquitous human herpes simplex virus HSV-1 (Table S1, [86]). Anthraquinone alterporriol Q (**15**) and the hydroanthraquinone tetrahydroaltersolanol C, obtained from a standing culture broth of *Alternaria* sp. ZJ-2008003, retrieved from *Sarcophyton* sp., showed antiviral activity against the porcine reproductive and respiratory syndrome virus (PRRSV), the most impacting infectious disease of pigs worldwide (Table S1 [87]). The polyketide–cyclopeptide (PKS-NRPS) metabolites (−) and (+)-pestaloxazine A (**16**) produced by *Pestalotiopsis* sp., also isolated from the octocoral *Sarcophyton* sp., showed antiviral activity against human *Enterovirus* 71 (EV71), the causative agent of hand, foot, and mouth disease (HFMD) that can lead to severe neurological complications in young children and infants [88]. 

### 3.4. Anticancer Activity

Cancer, characterized by the uncontrolled growth and spread of abnormal cells, is the second leading cause of death globally, accounting for 8.8 million fatal cases in 2015 (World Health Organization, http://www.who.int/cancer/en/). Since 1990, there has been a continuous increase in the number of anticancer compounds derived from marine sources that have progressed into preclinical and human clinical trials. Five marine invertebrate-derived compounds (at least) have been approved by the U.S. Food and Drug Administration (FDA) or the European Medicines Agency (EMA) as anticancer drugs [89]. Natural products from octocorals have a wide activity spectrum against several human cancer cell lines, including colon and lung adenocarcinoma, breast, cervix, hepatocellular and gingival carcinoma, prostate, gastric, and central nervous system cancer, and lymphocytic leukemia [90,91,92,93,94,95]. Since 2010, multiple bacterial and fungal associates from various octocoral species have been identified as producers of anticancer compounds with an equally ample activity spectrum (Table S1). 

For example, the soft-coral associated *Streptomyces* sp. OUCMDZ-1703 produces the chlorinated polyketides streptochloritide A and B (**17**) that possess cytotoxicity against breast cancer (MCF-7) cells (IC50 values of 9.9 and 20.2 μM, respectively) [96]. The ubiquinone-monoterpenoid derivative pseudoalteromone A (**18**), produced by *Pseudoalteromonas* sp. CGH2XX associated with *Lobophytum crissum*, is cytotoxic to human acute lymphoblastic leukemia (MOLT-4) cells, and shows anti-inflammatory activity by inhibiting the release of elastase by human neutrophils [97]. *Vibrio* sp. strain WMBA, obtained from the surface of *Sinularia polydactyla*, is the producer of several maleimide derivatives and aqabamycins (e.g., aqabamycin A (**3**)) that show, besides the antibacterial and antifungal activities mentioned above, cytotoxic effects against several cancer cell lines [59]. The basidiomycete fungus *Chondrostereum* sp., isolated from *Sarcophyton tortuosum*, is a rich source of hirsutane-framework sesquiterpenoids. However, its metabolic profile varies substantially depending on the composition of the culture medium [98]. In potato dextrose broth (PDB), the sesquiterpenoids chondrosterin A–E can be isolated, with chondrosterin A (**19**) showing significant cytotoxic activity against human lung carcinoma (A549), nasopharyngeal carcinoma (CNE-2), and human colon (LoVo) cancer lines, with IC50 values of 2.45, 4.95, and 5.47 μM, respectively [99]. In the same medium, the fungal associate also produces incarnal, which is cytotoxic towards eight different cancer cell lines [100]. When grown on glucose peptone yeast extract (GPY) medium prepared in seawater, hirsutanol A, E and F can be obtained. Hirsutanol A shows potent cytotoxicity against 15 different cancer cell lines, including human colon, lung, hepatic, nasopharyngeal, breast, and cervical cancer lines with IC50 values ranging from 0.58 to 8.27 µg/mL [101]. In general, IC50 values below 50 µg/mL are considered as active. Finally, when the fungus is cultured in liquid medium containing glycerol as carbon source, the sesquiterpenoid chondrosterin J can be harvested, which shows potent cytotoxic activities against nasopharyngeal carcinoma CNE-1 and CNE-2 cell lines, with IC50 values of 1.32 and 0.56 μM, respectively (Table S1). The cytotoxic effect of chondrosterin J is even stronger than those of chondrosterin A, hirsutanol A (CNE-1: 10.08 μM; CNE-2: 12.72 μM), and incarnal (CNE-1: 34.13 μM; CNE-2: 24.87 μM) [98]. This exemplifies that medium composition (and culture conditions) greatly impact the diversity (and quantity) of fungal secondary metabolites, highlighting the importance of the optimization of growth conditions for industrial upscaling. The ascomycete fungus *Alternaria* sp. ZJ-2008003, obtained from *Sarcophyton* sp., produces the anthraquinone alterporriol P, which is cytotoxic to human prostate adenocarcinoma (PC-3) and colon carcinoma (HTC-116) cells with IC50 values of 6.4 and 8.6 μM, respectively [87]. The ethyl acetate extract of an *Aspergillus* sp. strain isolated from *Dichotella gemmacea* exhibited significant cytotoxicity against a human lung carcinoma (A-549) cell line. Further investigation into this bioactive extract led to the identification of a new azaphilone derivative, aspergilone A (**20**), which exhibits cytotoxicity towards HL-60 human promyelocytic leukemia, MCF-7 human breast adenocarcinoma and A-549 human lung carcinoma cell lines [102]. Moreover, the chromones oxalicumone A (**21**) and B produced by *Penicillium oxalicum* isolated from *Muricella flexuosa* exhibit cytotoxicity against skin (A375) and colon (SW-620) cancer cells, whereby oxalicumone A is more potent [103].

### 3.5. Antimalarial, Anti-Inflammatory, Antineurodegenerative and Other Activities

Some protozoans, when transmitted to humans (generally via an animal vector), cause serious infectious diseases, such as malaria, sleeping sickness, Chagas’ disease, or toxoplasmosis, which pose a significant threat to global health, particularly in tropical and subtropical regions. Malaria develops upon being stung by a mosquito that carries a *Plasmodium*-type (protozoan) parasite (commonly *Plasmodium falciparum*) and is still a leading cause of morbidity and mortality in Africa, where 90% of all cases occur (WHO, July 2018, http://www.who.int/malaria/en/). Natural products play an important role in the eradication of malaria, and marine organisms are an excellent source of novel antiprotozoal compounds. However, reports on antiplasmodial or antiprotozoal activities of compounds derived from octocoral-associated microorganisms are still scarce. Yet one notable example is the tetronic acid nodulisporacid A (**22**), produced by the octocoral-associated ascomycete *Nodulisporium* sp. CRIF1, which exhibits promising activity against chloroquine-resistant *P. falciparum* strain 94, with IC50 values of 1–10 µM (Table S1 [104]).

Inflammation processes are correlated with the activation of the immune system in response to microbial infections, irritation or injury on tissues or organs. Pseudopterosins are diterpene glycosides retrieved from *Pseudopterogorgia elisabethae* (now *Antillogorgia elisabethae*) and rank among the first octocoral-derived compounds with described anti-inflammatory activity [2]. Pseudopterosin A has already undergone Phase II human clinical trials led by the Regents of the University of California [89], where it showed increased reepithelization and accelerated wound-healing processes [105]. In 2003, Mydlarz and colleagues demonstrated that the dinoflagellate symbiont *Symbiodinium* sp. is capable of synthesizing the Pseudopterosins A to D (Table S1) in physiologically significant levels, both from inorganic carbon and from the precursor geranylgeranyl-diphosphate [106], pointing out a symbiont origin of these anti-inflammatories. Proinflammatory enzymes, including the inducible nitric oxide synthase (iNOS), are key in the development of inflammatory diseases. Macrophages are among the leukocyte populations that can escalate the inflammation process and show enhanced expression of NOS enzymes. Therefore, in vitro anti-inflammatory activity is frequently assessed by the inhibition of the expression or production of iNOS proteins and by the inhibition of NO generation in macrophage cells [107]. An *Aspergillus terreus* strain isolated from *Sarcophyton subviride* is the producer of versicolactone B (**23**), a lactone that shows anti-inflammatory effects by inhibiting NO production in mouse macrophages ([108], see Table S1 for details). More lactones, including a variety of territrem derivatives, can be obtained from *Aspergillus terreus* strain SCSGAF0162, isolated from *Echinogorgia aurantiaca*. Some of these tremorgenic mycotoxins, including territrem D and E, show strong acetylcholinesterase (AChE) inhibitory activity with IC50 values of 4.2 and 4.5 nM, respectively. AChE inhibitors are currently among the most effective treatment targets for the design of drug candidates against Alzheimer’s disease, a neurodegenerative disorder that is the most common cause of dementia among the elderly [86]. 

Further interesting activities described for compounds from octocoral-associated microorganisms include antioxidant, insecticidal, and phytoregulatory effects. For example, *Aspergillus versicolor* LCJ-5-4, retrieved from the finger leather coral *Cladiella* sp., is the producer of tetraorcinol A (**24**), an orcinol with antioxidant activity, which functions as a scavenger against the 2,2-diphenyl-1-picrylhydrazyl (DPPH) radical [70]. Chrodrimanin B, a meroterpenoid composed of a sesquiterpenoid and a polyketide moiety, has been described for an *Aspergillus* sp. associated with the octocoral *Dichotella gemmacea* [109]. The compound exhibits insecticidal activity by blocking insect GABA-gated chloride channels [110]. The cyclopeptide-alkaloid spirotryprostatin F (**25**) from *Aspergillus fumigatus*, retrieved from *Sinularia* sp., shows phytoregulatory activity in low and ultralow doses, stimulating the growth of sprout roots of soy, corn, and buckwheat [111]. 

## 4. Genomic Insights into Natural Product Biosynthesis by Bacterial Symbionts of Octocorals 

**Figure 5:**
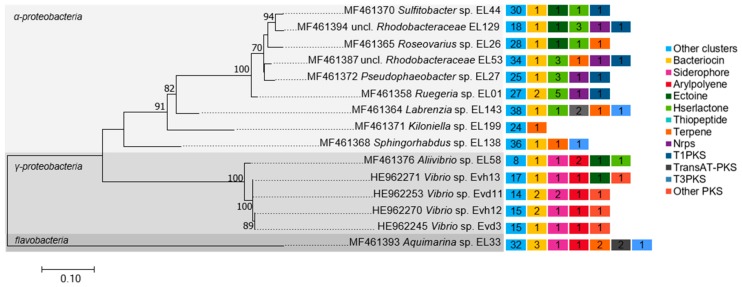
Secondary metabolite biosynthetic gene clusters (BGCs) from bacteria associated with octocorals. Right: the type and number of BGCs present on the genome sequences of 15 bacteria isolated from the gorgonian corals *Eunicella labiata* (EL) and *E. verrucosa* (Evh—healthy; Evd—diseased) are presented. Biosynthetic clusters were identified using the bacterial version of antiSMASH 3.0 [119]. Genome sequences are available under the accession numbers presented in the legend to Table 1. Left: 16S rRNA gene-based phylogenetic relationship of the 15 symbionts. The 16S rRNA gene tree was constructed in MEGA7 using the Maximum Likelihood method and General Time Reversible model with a discrete gamma distribution and invariable sites (GTR+G+I). Sequences were aligned using CLUSTALW algorithm prior to bootstrapped tests (100 repetitions) of phylogeny. Corresponding 16S rRNA gene-sequence accession numbers are given before each strain name.

Most culture collections of octocoral-associated bacteria published to date are rather small-sized, representing a reduced taxonomic diversity (consisting mostly of *Vibrio* spp.) usually retrieved from diseased coral tissue [112,113,114]. This and the general difficulty to captivate marine prokaryotes in the laboratory likely explain why reports on natural products from octocoral symbionts are still relatively limited. Recently, we developed an alternative strategy that enabled the cultivation of many dominant bacterial symbionts from the gorgonian coral *Eunicella labiata* [44], a prolific source of anticancer diterpenoids [5,115]. The isolated symbionts encompassed 13 classified and two unclassified bacterial genera within 7 bacterial families, including a variety of *Alphaproteobacteria* species. Genome sequencing of three such isolates, *Aquimarina* sp. strain EL33 [116], *Sphingorhabdus* sp. strain EL138 [117], and *Labrenzia* sp. strain EL143 [118], suggested versatile secondary metabolisms. Using antiSMASH v.3 [119], we have now mined 11 genomes representing different bacterial genera isolated from *E. labiata* by Keller-Costa et al. [44], in addition to four genomes from *Vibrio* strains associated with healthy and diseased *Eunicella verrucosa* specimens [120,121], for the presence of secondary metabolite biosynthetic genes clusters (BGCs). A total of 440 BGCs were found across the 15 genomes. These encompassed, primarily, terpene (*N* = 7), polyketide [PKS] (*N* = 14, i.e., 5× T1-PKS; 2× transAT-PKS; 3× T3-PKS; 4× other PKS), thiopeptide (*N* = 2), nonribosomal peptide [NRPS] (*N* = 4), bacteriocin (*N* = 15), arylpolyene (*N* = 7), and homoserine lactone [HSL] (*N* = 18) BGCs, besides many putative clusters (Figure 5). Terpene BGCs were found in the genomes of *Aquimarina* sp. EL33 (*Bacteroidetes*) and of the *Alphaproteobacteria* strains *Labrenzia* sp. EL143, *Sphingorhabdus* sp. EL138, *Kiloniella* sp. EL199, *Roseovarius* sp. EL26, and *Rhodobacteraceae* strain EL53 (closest genus *Phaeobacter*). Most of these terpenoid clusters do not present high homology to known clusters, highlighting the potential for the discovery of new natural products and biosynthetic pathways within these bacteria. Only the terpenoid gene cluster of *Sphingorhabdus* strain EL138 was found to share high homology with a known BGC, presenting 75% similarity with the gene cluster encoding the carotenoid astaxanthin [117]. This terpenoid is commercialized as a food dye, antioxidant, and nutritional supplement to prevent diabetes, cardiovascular diseases, neurodegenerative disorders, or cancer, and to stimulate immunization [122]. 

Besides BGCs prospection with antiSMASH, protein family (PFAM)-based analysis detected strictosidine synthase (EC 4.3.3.2) involved in monoterpenoid indole alkaloid biosynthesis on the genomes of several alphaproteobacterial strains (*Ruegeria* sp. EL01, *Rhodobacteraceae* strain EL53 and *Sphingorhabdus* sp. EL138) as well as on the *Aquimarina* sp. EL33 genome (Table 1). Additionally, *Sphingorhabdus* sp. EL138 and *Aquimarina* sp. EL33 possessed a limonene-1,2-epoxide hydrolase (EC 3.3.2.8) involved in monoterpene metabolism and degradation (Table 1). All 15 *Eunicella*-derived bacterial genomes possessed one or more genes for tri- and/or tetraterpenoid synthesis according to PFAM annotations. At least one squalene or phytoene synthase (EC 2.5.1.21/EC 2.5.1.32) gene involved in steroid or carotenoid synthesis was present on all the nine *Alphaproteobacteria* genomes and on the *Aquimarina* genome. Squalene epoxidase (PF08491.3) encoding genes for sterol biosynthesis were present on the genomes of *Labrenzia* sp. EL143, *Rhodobacteraceae* strain EL53, and all four *Vibrio* strains, while genes encoding for lycopene cyclases (PF05834.5) and hydroxyneurosporene synthase (PF07143.4) for carotenoid synthesis were found on the genomes of 10 of the 15 *Eunicella* associates (Table 1). This points to a widespread, yet uncharted capacity of octocoral bacterial associates to synthesize and metabolize terpenes, potentially contributing to the vast diversity of bioactive terpenoids characteristic of these animals. Furthermore, gene clusters involved in the biosynthesis of diverse polyketide synthases (PKS) and cyclases were noticeably present across all the inspected genomes (Table 1). AntiSMASH screening identified a polyketide BGC in seven out of nine *Alphaproteobacteria* associates (Figure 5). While the *Labrenzia* sp. EL143 genome contained a thiopeptide-trans-AT-PKS-NRPS hybrid and a T3-PKS cluster, all other strains in the *Rhodobacteraceae* family typically possessed a T1-PKS cluster. *Sphingorhabdus* sp. EL138 also possessed a putative T3-PKS gene cluster, whereas four PKS clusters were present on the *Aquimarina* genome, namely two T3-PKS and two trans-AT-PKS clusters. Thus, bacterial symbionts of octocorals are well-equipped for the synthesis of a wealth of polyketides, likely with pharmacologically relevant activities. Moreover, all the 15 genomes contained an ABM domain (PF03992.9) which is typically found in monooxygenases involved in the biosynthesis of several antibiotics (Table 1). *Aquimarina* sp. EL33 also harboured the lodAB operon responsible for the biosynthesis of marinocine [116], a lysin oxidase, antimicrobial protein that provokes cell death by generating hydrogen peroxide [123]. Our outcomes strengthen the notion of highly diversified secondary metabolisms among bacterial associates of octocorals, yet functional and comparative genomics approaches have seldom been applied to the study of octocoral-associated microorganisms. Type 2-PKS genes have been amplified from several *Actinomycetes* strains of the genera *Micromonospora* (**6**) and *Streptomyces* (**4**) isolated from the octocoral *Scleronephthya* sp. [124], and the authors also identified an analogue of the T2-PKS derived natural product jadomycin B, known for its cytotoxic and antibacterial activities [125], in the fermentation broth of Micromonospora strain A5-1.

### Genomic Insights into Biocatalysts

Industrial biocatalysis is a growing field with a wide range of applications, such as the biotreatment of waste and toxic chemicals, the improvement of food quality and its storage, the processing of several materials, such as paper and leather, and additives for detergents. The use of enzymes as catalysts is usually much cleaner and less hazardous than the use of chemical ones. Enzymes from marine organisms are frequently characterized by unique, habitat-related properties such as salt tolerance, hyper thermostability, barophilicity, cold adaptivity, or high pH tolerance [126], offering new types of biotechnological applications. While mining the 15 abovementioned bacterial genomes for the presence of BGCs, we noticed a wealth of genes encoding for the biosynthesis of several important biocatalysts, from chitinases to cellulases, amylases and a variety of proteases. This information has been compiled and discussed in Supplementary File S1 and Table S2. 

## 5. Optimization of Bioactive Compound Production to Meet Industrial Demands

**Figure 6:**
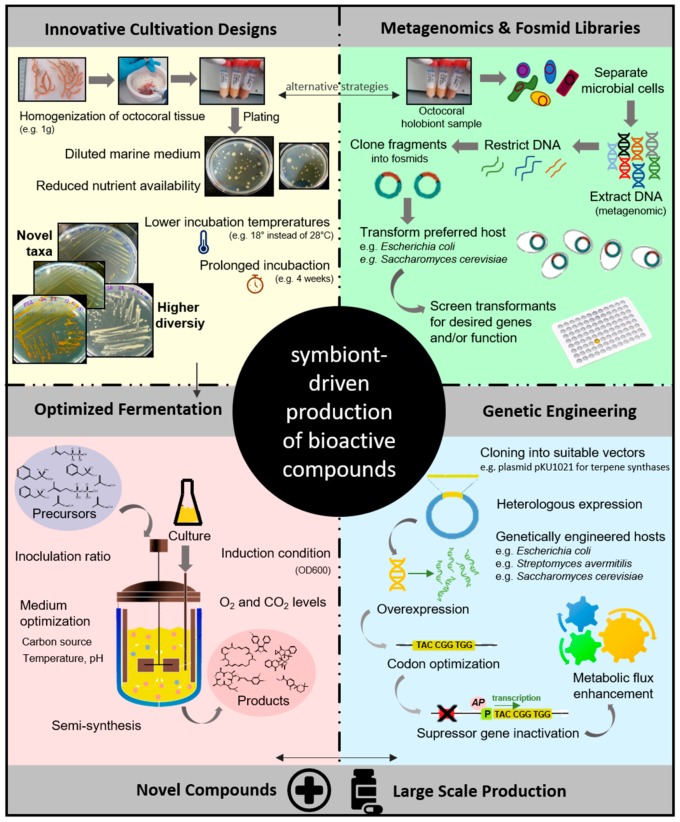
Optimal exploitation of the chemodiversity of octocoral-associated microbes. Schematic drawing illustrating important methodologies that need further development to widen the spectrum of bioactive natural products derived from octocoral-associated microorganisms and to optimize their production to meet industrial demands. Firstly, alternative cultivation approaches can increase the taxonomic and metabolic diversity of octocoral-associated microbes. Secondly, metagenomic tools and DNA-recombination technologies can provide access to valuable natural product biosynthetic gene clusters of often dominant symbionts recalcitrant to cultivation. Further, functional metagenomic studies can help to predict the symbionts’ dietary needs, aiding in the formulation of improved cultivation media. Genetic engineering and heterologous expression systems can be used to significantly scale-up natural product outputs. The optimization of fermentation processes ultimately improves natural product titer, either in the native producer strain or in the heterologous host.

As noticed above, the number of natural products discovered from marine microorganisms has sharply increased over the past decade, with octocorals being prosperous hosts of bacterial and fungal producers of secondary metabolites. This reservoir of bioactive microbes offers exciting alternatives to the often unprofitable and complicated chemical synthesis of natural products, and the unsustainable harvest of slow-growing and declining wild coral populations. Instead, microbial-derived bioactive compounds can be obtained from only tiny amounts (0.1–1 g) of coral material, being such quantities sufficient to establish culture collections of hundreds to thousands of bacterial or fungal associates. Efforts should therefore be directed towards the development and optimization of methodologies that lead to minimally invasive and economically reliable retrieval of bioactive secondary metabolites from the diverse and chemically complex microbial communities inhabiting octocorals. However, multiple challenges are to be met and overcome on the long path towards clinical trials and drug approval. In fact, the number of biotechnological processes described for large-scale production of marine, microbial-derived bioactive compounds is far from the sum of the newly discovered natural products [127]. Large pharmaceutical enterprises tend to be risk-aversive in their research and development programs and take up product-commercialization processes only at advanced stages. It is, thus, often left to research institutions and small spin-off and start-up companies to take on the early, risky steps of drug development, including production optimization, safety assessment, and first clinical trials [128,129]. To improve microbial-driven production processes, the following areas (summarized in Figure 6) need investment from research institutions and companies alike. At first, innovative cultivation strategies that expand the taxonomic and metabolic breath of cultivatable microbial communities should be sought. This would undoubtedly aid the continuous supply of novel bioactive compounds. The taxonomic variety of octocoral associated microbes present in culture collections is still quite small compared to the expected number of phylotypes as assessed by amplicon-based metagenomics surveys [22]. Because studies addressing the cultivatable fraction of the octocoral microbiome in a comprehensive fashion are scarce, the number of complete genome sequences from octocoral symbionts in public databases are prohibitively low in comparison with the broad range of symbiotic bacteria already well-characterized from other systems such as the marine sponge, rhizosphere, and human microbiomes. We have recently shown that the cultivatable fraction of octocoral-associated bacteria can be successfully expanded with relatively simple measures, such as reduced nutrient content in the culture medium, lower incubation temperatures, and prolonged incubation time [44]. Further success is expected from new medium formulations that contain specific nutrients envisaged to correspond to the dietary needs of symbiotic microorganisms. Here, functional metagenomics studies of octocoral microbiomes can help to predict the nutritional demands of symbionts, as valuable insights into abundant catabolic enzymes involved in nutrient metabolism can be gathered from shotgun sequencing projects. 

Once a “bioactive” octocoral symbiont has been permanently and successfully brought into laboratory culture, the challenge is to improve natural product yield. Biosynthesis of the desired bioactive compound by the natural producer strain usually occurs at a rate that is much lower than what is required for industrialization. Titer improvement in native producing strains can often be achieved by the careful optimization of fermentation processes, which includes adjusting the type of growth medium and carbon source, the temperature, pH, dissolved oxygen, and carbon dioxide levels. The inoculation ratio between the precursor substrate and carbon source and the inducer condition, i.e., inoculation time point and growth phase (OD600), are further important factors that require careful optimization [130]. In case that full fermentative processes are not economically viable or cannot be accomplished, semisynthetic strategies provide an alternative route in product development. Intermediate products are biologically synthesized before they are chemically converted to final products or, *vice versa*, a synthetic product is bio-converted using enzymes or fermentation processes into the end-product. For example, the *β*-lactam antibiotic cephalosporin C, derived from the marine ascomycete fungus *Acremonium chrysogenum*, was further developed by semi-synthesis into numerous commercial cephalosporin derivatives (e.g., cafalotin and cefazolin) with significantly increased antibiotic activity [127].

To meet the industrial demands of large-scale drug production, further genetic- and metabolic-engineering methodologies and strategies are often necessary. Frequently, the heterologous expression of the desired bioactive compound in genetically engineered microbial host cells, such as e.g., *Escherichia coli* or *Saccharomyces cerevisiae*, optimized for industrial-scale production, is preferred [130]. The synthase genes of interest are cloned into appropriate vectors which are then inserted into a suitable host cell to express the target genes. Diligent regulation of target gene expression using genetic approaches such as codon optimization and overexpression (e.g., through stronger promotors or transcriptional activators), as well as inactivation of repressor genes, have been very efficient in increasing natural-product titers [130]. A wide range of terpene synthases, for example, can be efficiently expressed in engineered *Streptomyces avermitilis* SUKA strains from which the native terpene synthase genes have been deleted. This way, the host is unable to produce endogenous terpenoid metabolites that could interfere with the production of heterologously expressed terpenoids. *Streptomyces* integrating plasmid vectors are used for the incorporation of genes encoding monoterpene, sesquiterpene, and diterpene synthases in *S. avermitilis* SUKA strains. Heterologous genes are inserted downstream of the appropriate synthase genes for precursor molecules, i.e., geranyl diphosphate synthase, farnesyl diphosphate synthase, or geranyl–geranyl diphosphate synthase under the control of a strong, constitutively expressed promoter (rpsJp) harbored in the integrating vector, e.g., pKU1021. The *S. avermitilis* SUKA system is suitable for the expression of a variety of terpenoid synthase genes from different bacterial phyla, including Gram-negative bacteria, and very useful for the discovery of new terpene synthases as well as the preparative isolation of terpenoid metabolites [13]. More examples of suitable heterologous expression systems and engineering strategies for optimized natural product production are given in the extensive reviews on microbial metabolic engineering and marine fungi biotechnology by Park et al. [130] and Silber et al. [127], respectively.

The combination of heterologous expression systems and modern metagenomics tools makes it possible that even the “pristine” resource of yet uncultivatable octocoral symbionts, be it bacteria, fungi, or other microeukaryotes, can be approached. Metagenomic libraries created *via* next generation-sequencing technologies give access to the total genomic pool of nature’s microbiomes without the need of cultivation. In complex host–microbe systems, it bears great potential in the exploitation of the metabolism of often dominant bacterial symbionts recalcitrant to cultivation and to their concealed chemodiversity. High molecular-weight fragments of metagenomic DNA are ligated into fosmid, cosmid, or BAC vectors and, e.g., *Escherichia coli* transformants are then screened for a given bioactivity. So-called clone super-pooling strategies allow the efficient screening of 10,000 and more fosmid clones [131]. The combination of metagenomics and heterologous systems enables industrial-scale production of compounds never isolated and produced by so-far uncultivatable microorganisms [132]. Yet, despite the astonishing progress in molecular biology made in the last 20 years or so, the application of this technique in large-scale production of natural products is still scarce [49]. Most challenging is the complexity and size of the genetic clusters encoding for most natural products, and their intricate genetic regulation involving multiple regulatory cascades and networks [127]. Certainly, for marine microbial-derived natural products, the above-presented approaches are still in their infancy, yet investment in biotechnology development is the path for a sustainable production of octocoral- and other marine-derived microbial metabolites. 

## 6. Concluding Remarks

This review demonstrates that the potential of octocoral-associated microbes to serve as prolific sources of novel natural products of interest in applied biotechnology is indisputable. However, much, if not all, of the chemodiversity known so far for these symbionts derives from bacterial and fungal cultures isolated in the laboratory. Still, many symbionts captured in culture have not been thoroughly explored in terms of their secondary metabolism and natural-product biosynthesis capacities. To this end, comprehensive genome mining coupled to laboratory experimentation needs to be applied for the already quite diverse panel of cultivatable octocoral symbionts, with apparently promising discoveries to be made if we are to mine the metabolism of cultivatable fungi and bacteria in depth. Quite clearly, it is imperative that we enlarge our analytical toolbox to integrate the wealth of recently developed metagenomics and next-generation sequencing technologies to the study of the complex microbiomes of octocorals. Although these approaches have been successfully implemented in biodiversity surveys, metagenomics-assisted investigation of natural product biosynthesis by uncultured octocoral symbionts awaits further development. Dedicated, cultivation-independent experiments hold promise in illuminating the metabolism of a wide diversity of not cultured or hitherto uncultivatable octocoral symbionts and, will be fundamental to allow the identification of microbial gene clusters involved in the biosynthesis of octocoral-derived metabolites in a comprehensive fashion. If coupled to heterologous expression systems fine-tuned to promote the biosynthesis of specific compounds (which is the case of existing terpenoid production platforms by surrogate bacterial hosts), metagenomics-guided metabolite biosynthesis by octocoral symbionts (from DNA extraction to recombination and expression) could become an alternative route to sustainably harvest novel natural products from lesser-known and hard-to-culture microorganisms. Such a multidisciplinary undertaken is key to achieving a sustainable response to the urgent industrial demand for novel drugs and enzyme varieties and can only be fostered through increased investment into next-generation biopharmaceuticals research and development, which considers well-grounded and environmentally reasonable exploitation of natural resources.

## Figures and Tables

**Table 1:** Protein families (PFAM) and/or cluster of orthologous groups (COGs)-based annotation of terpene, polyketide, and antibiotic encoding genes present on the genomes of 15 octocoral (*Eunicella labiata* and *E. verrucosa*) associated bacteria (*Alphaproteobacteria* (*N* = 9), *Gammaproteobacteria* (*N* = 5), and *Bacteroidetes* (*N* = 1)).

Category	Accession	Name	Description	Number of Open Reading Frames Detected per Gorgonian-Associated Bacterial Genome														
				EL01	EL26	EL27	EL44	EL53	EL129	EL143	EL138	EL199	EL58	EL33	Evd3	Evd11	Evh12	Evh13
Monoterpene synthesis and metabolism	PF03088.9	Str_synth	Strictosidine synthase	1	0	0	0	2	0	0	2	0	0	3	0	0	0	0
	PF07858.5	LEH	Limonene-1,2-epoxide hydrolase catalytic domain	0	0	0	0	0	0	0	3	0	0	1	0	0	0	0
Tri- and tetraterpene synthesis	PF00494.12/COG1562	SQS_PSY/ERG9	Squalene/phytoene synthase	1	1	1	1	1	1	1	2	1	0	1	0	0	0	0
	COG1233	COG1233	Phytoene dehydrogenase and related proteins	0	0	1	2	0	0	1	2	0	0	3	0	0	0	0
	PF08491.3	SE	Squalene epoxidase	0	0	0	0	1	0	1	0	0	0	0	1	1	1	1
	PF05834.5	Lycopene_cycl	Lycopene cyclase protein	0	0	0	0	0	0	0	1	0	3	1	0	0	0	0
	PF07143.4	CrtC	Hydroxyneurosporene synthase	1	0	1	0	1	0	0	0	0	1	0	2	2	2	2
Polyketide synthases	PF08392.5	FAE1_CUT1_RppA	FAE1/Type III polyketide synthase-like protein	0	1	0	0	1	1	0	1	1	0	0	0	0	0	0
	PF00195.12	Chal_sti_synt_N	Chalcone and stilbene synthases, N-terminal domain	0	0	1	1	1	0	1	1	0	0	2	0	0	0	0
	PF02797.8	Chal_sti_synt_C	Chalcone and stilbene synthases, C-terminal domain	0	0	0	0	0	0	1	1	0	0	0	0	0	0	0
	COG2761	FrnE	Predicted dithiol-disulfide isomerase involved in polyketide biosynthesis	2	1	1	1	2	1	1	2	2	1	1	1	2	2	1
	COG3321	COG3321	Polyketide synthase modules and related proteins	1	0	1	1	1	1	0	11	0	0	11	2	1	1	1
	COG5285	COG5285	Protein involved in biosynthesis of mitomycin antibiotics/polyketide fumonisin	2	1	2	1	1	1	0	2	1	0	0	0	0	0	0
Polyketide cyclases	PF03364.13	Polyketide_cyc	Polyketide cyclase/dehydrase and lipid transport	1	1	1	1	1	1	1	0	1	1	0	1	1	1	1
	PF10604.2	Polyketide_cyc2	Polyketide cyclase/dehydrase and lipid transport	5	3	2	4	1	4	8	3	0	0	5	0	0	0	0
	PF07366.5	SnoaL	SnoaL-like polyketide cyclase	26	8	8	11	7	5	20	9	1	0	16	15	6	15	2
Nonribosomal peptides	PF08415.3	NRPS	Nonribosomal peptide synthase	2	0	0	0	0	0	0	0	0	0	0	0	0	0	0
Amino-glycoside antibiotics	PF02522.7	Antibiotic_NAT	Aminoglycoside 3-*N*-acetyltransferase	0	0	0	0	0	0	0	0	0	0	1	1	0	0	0
	PF03992.9	ABM	Antibiotic biosynthesis monooxygenase	11	9	5	2	2	4	11	9	6	2	5	6	6	6	6
Polycyclic peptide antibiotics	PF04737.6	Lant_dehyd_N	Lantibiotic dehydratase, N-terminus	0	0	0	0	0	0	0	0	0	0	2	0	0	0	0
	PF04738.6	Lant_dehyd_C	Lantibiotic dehydratase, C-terminus	0	0	0	0	0	0	0	0	0	0	1	0	0	0	0

For each (amino acid-translated) bacterial genome, PFAMs and COGs were predicted using the WebMGA platform (http://weizhong-lab.ucsd.edu/metagenomic-analysis/) and COG and PFAM entries relevant for terpene, polyketide, and/or antibiotic biosynthesis are shown. All “EL” strains have been isolated from the gorgonian coral *Eunicella labiata* as described in reference [44]. All “Ev” strains have been obtained from the gorgonian coral *Eunicella verrucosa* as described in reference [121]. “Evh” strains derive from healthy, while “Evd” strains derive from necrotic *E. verrucosa* tissue. Bacterial strain names and corresponding genome sequence accession numbers are as follows: *Ruegeria* sp. EL01 (OMPS01000001-OMPS01000049); *Roseovarius* sp. EL26 (OUMZ01000001-OUMZ01000007); *Pseudophaeobacter* sp. EL27 (OMPQ01000001-OMPQ01000016); *Sulfitobacter* sp. EL44 (OMPT01000001-OMPT01000014); *Rhodobacteraceae* strain EL53 (OMPR01000001-OMPR01000019); *Rhodobacteraceae* strain EL129 (ONZJ01000001-ONZJ01000003); *Labrenzia* sp. EL143 (OGUZ01000001-OGUZ01000034 [118]; *Sphingorhabdus* sp. EL138 (OGVD01000001-OGVD01000004 [117]); *Kiloniella* sp. EL199 (OMPU01000001-OMPU01000019); *Aliivibrio* sp. EL58 (OMPC01000001-OMPC01000012); *Aquimarina* sp. EL33 (FLRG01000001-FLRG01000020 [116]; *Vibrio* sp. strain Evd3 (ORXW01000001-ORXW01000013); *Vibrio* sp. Evd11 (OSDX01000001-OSDX01000117); *Vibrio* sp. Evh12 (FAUO01000001-FAUO01000017 [120]); *Vibrio* sp. Evh13 (OSDW01000001-OSDW01000043).

## Supplementary Materials

The following files are available online at http://www.mdpi.com/1660-3397/16/12/485/s1: Supplemental File S1. Biocatalysts from microbial symbionts of octocorals. Further information and literature on biocatalyst activities described for octocorals and their microbial associates, together with the genome mining results of 15 *Eunicella* spp.-derived bacterial genomes for the presence of BGCs (see also Table S2). The latter revealed a wealth of genes encoding for the biosynthesis of several important biocatalysts, from chitinases to cellulases, amylases, and a variety of proteases, Table S1. Bioactive natural products produced by octocoral associated microorganisms. Inventory of >80 bioactive compounds produced by bacteria and fungi associated with octocorals, Table S2. Copy number of open reading frames (ORFs) of biocatalyst protein families (PFAM) present on the genomes of 15 octocoral-associated bacteria.
