# Supplementary material for: Bioactive Secondary Metabolites from Octocoral-Associated Microbes—New Chances for Blue Growth
Source: Mar Drugs. 2018 Dec 4;16(12):485. doi: 10.3390/md16120485 (PMC6316421; doi:10.3390/md16120485)
Supplement: Supplementary file 1 [file marinedrugs-16-00485-s001.zip › Supplementary_revised/Supplementary File S1.docx]

# **Supplementary File S1 - Biocatalysts from microorganisms associated with octocorals**

Although marine biocatalyst resources are represented by microbes, algae, plants and animals, most bioprospecting activity is indeed focused on microbial products and much effort is directed towards extremophiles and symbiotic microorganisms [1]. Suspension-feeding marine invertebrates such as octocorals can be regarded as true “samplers” of both marine particles and microbes, with their distinct and resident microbial communities functioning as natural bioreactors that mediate biogeochemical cycling and recalcitrant compound degradation in the benthic zone [2]. Several biocatalyst activities have been described for octocorals and their associated microbiome, including amylase, protease, chitinase and carboxymethyl-cellulase activity. However, biocatalyst activities in this host-microbial interaction are still largely underexplored. Amylases, the enzymes that hydrolyse starch molecules into low molecular weight products composed of glucose units, are produced by a wide range of bacteria and fungi. Microbial enzymes are more stable than both plant- and animal-derived amylases, offering wider industrial applications [3]. Thus far, *Bacillus* is the most used genus for the commercial production of heat-stable amylases [1]. *Bacillus aquimaris* strain MKSC 6.2, isolated from, *Sinularia* sp., is an efficient degrader of both soluble and granular starch. Its partially purified α-amylase shows the ability to degrade raw corn, rice, sago, cassava, and potato starches with adsorption percentage in the range of 65–93% [4]. Cellulose, the most abundant, renewable organic polymer on the planet and a dominant waste material of agriculture, is degraded by cellulases into cello-oligosaccharides, cellobiose and glucose. A range of marine bacteria and fungi produce cellulases [5,6] and cellulase activity was detected in a metagenomic fosmid library of microorganisms associated with the scleractinian coral *Siderastrea stellata*. The library comprised 3552 clones and six of them were positive for cellulolytic activity [7]. Bacterial isolates of the genera *Aeromonas,* *Pseudomonas*, *Flavobacterium*, *Micrococcus*, and *Kurthia*, obtained from the octocoral *Lobophython* sp., all show *in-vitro* amylase and cellulase activities. Further, all except *Kurthia* also have detectable activities of proteases, which hydrolyse the peptide bonds in proteins [8]. Chitin, the polymer of (1→4)-β-linked N-acetylglucosamine (GlcNAc), is the second most abundant natural polymer on earth after cellulose, and the most abundant one in the marine environment. Chitin-degrading enzymes, chitinases, have an array of applications in the food, medical and agricultural sector as they can be used in seafood waste processing, bioplastic production, fungal pathogen control or in adhesives for wound healing [9,10]. Crude extracts obtained from the sea fan *Gorgonia ventalina* contain detectable levels of exochitinase (EC 3.2.1.52) activity [11]. Genes predicted to encode proteins similar to cellulases and chitinases are predicted in the genome of the scleractinian coral Acropora digitifera and cellulose- and chitin-degrading enzyme activities can be measured in the protein extracts of seven scleractinian coral species (A. digitifera, Galaxea fascicularis, Goniastrea aspera, Montipora digitata, Pavona divaricata, Pocillopora damicornis and Porites australiensis) [12], suggesting a wide-spread occurrence of cellulases and chitinases in coral holobionts. It is known indeed from a range of marine bacterial species that they produce chitinases to feed on chitin as a sole nutrient source [13]. PFAM-based analysis of 15 bacterial genomes derived from the gorgonian corals *Eunicella labiata* and *E. verrucosa* revealed that *Aquimarina* sp. strain EL33 and all four *Vibrios* strains carry indeed ORFs for amylases, chitinase and cellulases while the genome of *Aliivibrio* sp. strain EL58 possesses amylase and chitinase encoding genes (**Table S2**). An earlier RAST (Rapid Annotation using Subsystem Technology)-based survey of *Aquimarina* sp. strain EL33 had detected nineteen endochitinase (EC 3.2.1.14) -encoding genes on the strain’s genome, suggesting that this bacterial associate could be an efficient chitin degrader [14]. Conversely, chitinase, amylase and cellulase encoding genes seem to be largely absent in most of the alphaproteobacterial associates inspected in this review. An exception is *Labrenzia* sp. strain EL143, for which amylase and cellulase ORFs were detected with PFAM-based annotation, while RAST-based annotation revealed the presence of an endochitinase (EC 3.2.1.14) and three exochitinases (EC 3.2.1.52) [15]. Three of the four *Vibrio* strains from *E. verrucosa* also harboured genes encoding for an alginate lyase, an enzyme that breaks down the gelling polysaccharide alginate produced by brown algae (*Phaeophyceae*), as well as some heterotrophic bacteria of the families *Pseudomonadaceae* and *Azotobacteriaceae*. Potential application of alginate lyases include the treatment of infections caused by *P. aeruginosa* mucoid colonies [16] and the manufacturing of wound dressings. Common to all 15 gorgonian-derived bacterial genomes is the presence of protease encoding genes, with *Aquimarina* sp. EL33 having the highest number of protease-related ORFs (**Table S2**). Overall, these genomic insights suggest versatile biocatalytic capabilities of gorgonian coral associated microbes, worthwhile for further exploration.

**References**

1. Trincone, A. Marine biocatalysts: Enzymatic features and applications. *Marine Drugs* **2011**, *9*, 478-499, doi:10.3390/md9040478.

2. Hentschel, U.; Usher, K.M.; Taylor, M.W. Marine sponges as microbial fermenters. *Fems Microbiology Ecology* **2006**, *55*, 167-177, doi:10.1111/j.1574-6941.2005.00046.x.

3. De Souza, P.M.; Magalhães, P.O. Application of microbial α-amylase in industry - A review. *Brazilian Journal of Microbiology* **2010**, *41*, 850-861, doi:10.1590/S1517-83822010000400004.

4. Puspasari, F.; Nurachman, Z.; Noer, A.S.; Radjasa, O.K.; van der Maarel, M.J.E.C.; Natalia, D. Characteristics of raw starch degrading a-amylase from *Bacillus aquimaris* MKSC 6.2 associated with soft coral *Sinularia* sp. *Starch* **2011**, *63*, 461–467, doi:10.1002/star.201000127.

5. Fu, X.; Liu, P.; Lin, L.; Hong, Y.; Huang, X.; Meng, X.; Liu, Z. A novel endoglucanase (Cel9P) from a marine bacterium *Paenibacillus* sp. BME-14. *Applied Biochemistry and Biotechnology* **2010**, *160*, 1627-1636, doi:10.1007/s12010-009-8648-2

6. Zhang, C.; Kim, S.-K. Research and application of marine microbial enzymes: status and prospects. *Marine Drugs* **2010**, *8*, 1920-1934, doi:10.3390/md8061920.

7. Sousa, F.M.O.; Moura, S.R.; Quinto, C.A.; Dias, J.C.T.; Pirovani, C.P.; Rezende, R.P. Functional screening for cellulolytic activity in a metagenomic fosmid library of microorganisms associated with coral. *Genetics and Molecular Research* **2016**, *15*, gmr.1504877, doi:10.4238/gmr.15048770.

8. Mohapatra, B.R.; Bapuji, M.; Sree, A. Production of industrial enzymes (amylase, carboxymethylcellulase and protease) by bacteria isolated from marine sedentary organisms. *Acta Biotechnologica* **2003**, *23*, 75 –84, doi:10.1002/abio.200390011.

9. Beier, S.; Bertilsson, S. Bacterial chitin degradation—mechanisms and ecophysiological strategies. *Frontiers in Microbiology* **2013**, *4*, 149, doi:10.3389/fmicb.2013.00149.

10. Fukamizo, T. Chitinolytic enzymes catalysis, substrate binding, and their application. *Current Protein and Peptide Science* **2000**, *1*, 105-124.

11. Douglas, N.L.; Mullen, K.M.; Talmage, S.C.; Harvell, C.D. Exploring the role of chitinolytic enzymes in the sea fan coral, *Gorgonia ventalina*. *Marine Biology* **2007**, *150*, 1137-1144, doi:10.1007/s00227-006-0444-8.

12. Yoshioka, Y.; Tanabe, T.; Iguchi, A. The presence of genes encoding enzymes that digest carbohydrates in coral genomes and analysis of their activities. *PeerJ* **2017**, *5*, e4087, doi:10.7717/peerj.4087.

13. Osawa, R.; Koga, T. An investigation of aquatic bacteria capable of utilizing chitin as the sole source of nutrients. *Letters in Applied Microbiology* **1995**, *21*, 288-291, doi:10.1111/j.1472-765X.1995.tb01062.x.

14. Keller-Costa, T.; Silva, R.; Lago-Lestón, A.; Costa, R. Genomic insights into *Aquimarina* sp. EL33, a bacterial symbiont of the gorgonian coral *Eunicella labiata*. *Genome Announcements* **2016**, *4*, e00855-00816, doi:10.1128/genomeA.00855-16.

15. Rodrigues, G.N.; Lago-Lestón, A.; Costa, R.; Keller-Costa, T. Draft genome sequence of *Labrenzia* sp. strain EL143, a coral-associated alphaproteobacterium with versatile symbiotic living capability and strong halogen degradation potential. *Genome Announcements* **2018**, *6*, e00132-00118, doi:10.1128/genomeA.00132-18

16. Bugli, F.; Posteraro, B.; Papi, M.; Torelli, R.; Maiorana, A.; Sterbini, F.P.; Posteraro, P.; Sanguinetti, M.; De Spirito, M. In vitro interaction between alginate lyase and amphotericin B against *Aspergillus* *fumigatus* biofilm determined by different methods. *Antimicrobial Agents and Chemotherapy* **2013**, *57*, 1275-1282, doi:10.1128/AAC.01875-12.
