# Supplementary material for: Bioactive Secondary Metabolites from Octocoral-Associated Microbes—New Chances for Blue Growth
Source: Mar Drugs. 2018 Dec 4;16(12):485. doi: 10.3390/md16120485 (PMC6316421; doi:10.3390/md16120485)
Supplement: Supplementary file 1 [file marinedrugs-16-00485-s001.zip › Supplementary_revised/Table S1.docx]

**Table S1.** Bioactive natural products produced by octocoral associated microorganisms.

| **Microorganism** | **Host species and family** | **Compound and chemical class** | **Bioactivity** | **Reference** |
| --- | --- | --- | --- | --- |
| *Bacteria* |  |  |  |  |
| ***Bacillus amyloliquefaciens*** Firmicutes Bacilli | *Junceella juncea* Ellisellidae | **macrolactin V**  **(2)**  macrolide | strongly antibacterial activity against *Escherichia. coli*, *Bacillus subtilis*, and *Staphylococcus aureus* | [1] |
| ***Pseudoalteromonas* sp.** Proteobacteria γ-proteobacteria | *Leptogorgia alba* Gorgoniidae | **alteramide A**  **(1)**  polyketide | antifungal activity | [2] |
| ***Pseudoalteromonas* sp.** Proteobacteria γ-proteobacteria | *Lobophytum crassum* Alcyoniidae | **pseudoalteromones A & B (18)** ubiquinone-monoterpenoids | anti-inflammatory activity - inhibition of release of elastase by human neutrophils; pseudoalteromone A also shows anticancer activity against MOLT-4 and T-47D cells | [3,4] |
| ***Streptomyces* sp.** Actinobacteria | unidentified soft coral (China) | **strepchloritide A & B**  **(17)**  polyketides | antibacterial activity against *S. aureus*; anticancer activity against MCF-7 cells | [5] |
| ***Vibrio* sp.** Proteobacteria  γ-proteobacteria | *Sinularia polydactyla* Alcyoniidae | **aqabamycin A–G**  **(3)**  maleimids | antibacterial, antifungal and anticancer activities | [6] |
| *Fungi* |  |  |  |  |
| ***Alternaria sp*.** Ascomycota Dothideomycetes | *Sarcophyton* sp. Alcyoniidae | **tetrahydroaltersolanol C** anthraquinone | active against the porcine reproductive and respiratory syndrome virus (PRRSV) | [7] |
| ***Alternaria sp.*** Ascomycota Dothideomycetes | *Sarcophyton* sp. Alcyoniidae | **alterporriols P & Q**  **(15)**  anthraquinones | alterporriol Q has antiviral activity against PRRSV; alterporriol P is anticancerous against PC-3 and HTC-116 | [7] |
| ***Aspergillus elegans*** Ascomycota Eurotiomycetes | *Sarcophyton* sp. Alcyoniidae | **4'-O-methoxyasperphenamate (4)**  pheylalanine derivative | antibacterial activity against *Staphylococcus epidermidis* | [8] |
| ***Aspergillus elegans*** Ascomycota Eurotiomycetes | *Sarcophyton* sp. Alcyoniidae | **asperphenamate** pheylalanine derivative | antibacterial activity against *S. epidermidis* | [8] |
| ***Aspergillus elegans*** Ascomycota Eurotiomycetes | *Sarcophyton* sp. Alcyoniidae | **aspochalasin D, I, J & H**  **(12)**  alkaloids / cytochalasins | strong antifouling activity against larval settlement of the barnacle *Balanus amphitrite* | [8] |
| ***Aspergillus fumigatus*** Ascomycota Eurotiomycetes | *Sinularia* sp. Alcyoniidae | **spirotryprostatin F**  **(25)**  cyclopetide-alkaloid | stimulatory phytoregulatory activity on sprout roots of soy, corn and buckwheat | [9] |
| ***Aspergillus ochraceus*** Ascomycota Eurotiomycetes | *Dichotella gemmacea* Ellisellidae | **ochralate A** pyrazinone derivative | antibacterial activity against *Enterobacter aerogenes* | [10] |
| ***Aspergillus ochraceus***  Ascomycota Eurotiomycetes | *Dichotella gemmacea* Ellisellidae | **ochromide B**  pyrazinone derivative | antibacterial activity against *E. aerogenes* | [10] |
| ***Aspergillus* sp.** Ascomycota Eurotiomycetes | *Muricella abnormalis* Acanthagorgiidae | **asperpeptide A**  **(5)**  cyclopeptide | antibacterial activity against *Bacillus cereus* and *S. epidermidis* | [11] |
| ***Aspergillus* sp.** Ascomycota Eurotiomycetes | *Muricella abnormalis* Acanthagorgiidae | **22-O-(NMe-L-valyl)-21-epi-aflaquinolone B**  **(14)**  hydroquinolone | very potent antiviral activity against respiratory syncytial virus (RSV). | [12] |
| ***Aspergillus* sp.** Ascomycota Eurotiomycetes | *Melitodes squamata* Melithaeidae | **aspergillipeptide C**  **(13)**  cyclopeptide | strong antifouling activity against *Bugula neritina* larvae settlement | [13] |
| ***Aspergillus* sp.** Ascomycota Eurotiomycetes | *Dichotella gemmacea* Ellisellidae | **aspergilone A**  **(20)**  benzylazaphilones | anticancer activity against HL-60, MCF-7 and A-549; potent antifouling activity against *B. amphitrite* larvae settlement | [14] |
| ***Aspergillus* sp.** Ascomycota Eurotiomycetes | *Dichotella gemmacea* Ellisellidae | **8-O-methylnidurufin** anthraquinone | antibacterial activity against *Micrococcus luteus* | [15] |
| ***Aspergillus* sp.** Ascomycota Eurotiomycetes | *Melitodes squamata* Melithaeidae | **9a,14-dihydroxy-6b-p-nitrobenzoylcinnamolide** alkaloid | strong antiviral activity against influenza virus H1N1 and H3N2 | [16] |
| ***Aspergillus* sp.** Ascomycota Eurotiomycetes | *Melitodes squamata* Melithaeidae | **7a,14-dihydroxy-6b-p-nitrobenzoylconfertifolin** alkaloid | moderate antiviral activit against H1N1 and H3N2 | [16] |
| ***Aspergillus* sp.** Ascomycota Eurotiomycetes | *Melitodes squamata* Melithaeidae | **aspergilliamide** aspergillic acid | weak toxicity to brine shrimp *Artemia salina* | [17] |
| ***Aspergillus* sp.** Ascomycota Eurotiomycetes | *Melitodes squamata* Melithaeidae | **ochratoxin A butyl ester** ochratoxin | significant toxicity to *Artemia salina* | [17] |
| ***Aspergillus* sp.** Ascomycota Eurotiomycetes | *Melitodes squamata* Melithaeidae | **5-hydroxy-3-isopropyl-4-methoxyfuranone**  γ-lactones | significant toxicity to *Artemia salina* | [18] |
| ***Aspergillus* sp.** Ascomycota Eurotiomycetes | *Dichotella gemmacea* Ellisellidae | **chrodrimanin B** terpenoid | insecticidal activity: potent blockers of insect GABA-gated chloride channels | [19,20] |
| ***Aspergillus* sp.** Ascomycota Eurotiomycetes | *Dichotella gemmacea* Ellisellidae | **methyl sydowate (7)** sesquiterpenoid | modest antibacterial activity against *S. aureus* | [21] |
| ***Aspergillus sydowii*** Ascomycota Eurotiomycetes | *Verrucella umbraculum* Ellisellidae | **fumiquinazoline D** indole alkaloid | antifouling activity towards bryozoan *Bugula neritina* larvae settlement | [22] |
| ***Aspergillus sydowii*** Ascomycota Eurotiomycetes | *Verrucella umbraculum* Ellisellidae | **cyclotryprostatin B** indole alkaloid | antifouling activity towards *B. neritina* larvae settlement | [22] |
| ***Aspergillus terreus*** Ascomycota Eurotiomycetes | *Sinularia kavarattiensis* Alcyoniidae | **aspernolide A** aromatic butenolid | mild anticancer activity towards H460, ACHN, Calu, Panc1 and HCT-116 cell lines | [23] |
| ***Aspergillus terreus*** Ascomycota Eurotiomycetes | *Echinogorgia aurantiaca* Plexauridae | **asperterrestide A** cyclopeptide | antiviral activity against influenza virus strains H1N1 and H3N2; anticancer activity against human tumor cell lines | [24] |
| ***Aspergillus terreus*** Ascomycota Eurotiomycetes | *Echinogorgia aurantiaca* Plexauridae | **territrem D and E** lactones / territrem derivatives | territrem D shows potent antifouling activity against *B. amphitrite* larvae settlement and, together with territrem E, strong anti-neurodegenerative activity (acetylcholinesterase (AChE) inhibitors) | [25] |
| ***Aspergillus terreus*** Ascomycota Eurotiomycetes | *Echinogorgia aurantiaca* Plexauridae | **11a-dehydroxy-isoterreulactone A** lactone / territrem derivative | antiviral activity towards HSV-1 | [25] |
| ***Aspergillus terreus*** Ascomycota Eurotiomycetes | *Echinogorgia aurantiaca* Plexauridae | **isobutyrolactone II** lactone / butyrolactone derivative | antiviral activity towards HSV-1 | [25] |
| ***Aspergillus terreus*** Ascomycota Eurotiomycetes | *Sarcophyton subviride* Alcyoniidae | **versicolactone B**  **(23*)**  lactone | antiinflammatory activity against nitric oxide (NO) production in RAW264.7 mouse macrophages | [26] |
| ***Aspergillus versicolor***  Ascomycota Eurotiomycetes | *Dichotella gemmacea* Ellisellidae | **aroyl uridine derivative 1, 2**  **(6)**  nucleosides | antibacterial activity against *S. epidermidis* and toxicity towards brine shrimp *A. salina* | [27] |
| ***Aspergillus versicolor***  Ascomycota Eurotiomycetes | *Cladiella* sp. Alcyoniidae | **tetraorcinol A**  **(24)**  phenol / orcinol | weak antioxidant activity/radical scavenger against the 1,1-diphenyl-2-picrylhydrazyl (DPPH) radical | [28] |
| ***Aspergillus versicolor*** Ascomycota Eurotiomycetes | *Cladiella* sp. Alcyoniidae | **cottoquinazoline D**  **(11)**  alkaloid | modest antifungal activity against *Candida albicans* | [29] |
| ***Chondrostereum* sp.** Basidiomycota Agaricomycetes | *Sarcophyton tortuosum*  Alcyoniidae | **chondrosterin J** sesquiterpenoid | potent anticancer activity y towards CNE-1 and CNE-2 cell lines | [30] |
| ***Chondrostereum* sp.** Basidiomycota Agaricomycetes | *Sarcophyton tortuosum*  Alcyoniidae | **chondrosterin A**  **(19)**  sesquiterpenoid | anticancer activity towards A549, CNE-2 and LoVo cancer cells | [31] |
| ***Chondrostereum* sp.** Basidiomycota Agaricomycetes | *Sarcophyton tortuosum*  Alcyoniidae | **incarnal**  sesquiterpenoid | potent anticancer activity against eight different cancer cell lines | [32] |
| ***Chondrostereum* sp.** Basidiomycota Agaricomycetes | *Sarcophyton tortuosum*  Alcyoniidae | **hirsutanol A**  sesquiterpenoid | potent anticancer activity is against 15 different cancer cell lines | [33] |
| ***Cochliobolus lunatus*** Ascomycota Dothideomycete | *Dichotella gemmacea* Ellisellidae | **cochliomycin A**  lactone | strong antifouling activity against *B. amphitrite* larvae settlement and moderate antibacterial activity against *S. aureus* | [34] |
| ***Nodulisporium* sp.** Ascomycota Sordariomycetes | unidentified soft coral (Thailand) | **nodulisporacid A**  **(22)**  lactone / tetronic acid | antimalarial activity against chloroquine resistant *Plasmodium falciparum* | [35] |
| ***Penicillium citrinum***  Ascomycota Eurotiomycetes | *Annella sp.* Supergorgiidae | **penicillanthranin A**  **(8)**  anthraquinone | antibacterial activity against *S. aureus* and methicillin resistant *S. aureus* (MRSA) | [36] |
| ***Penicillium commune*** Ascomycota Eurotiomycetes | *Muricella abnormalis* Acanthagorgiidae | **communols A–G**  **(9)**  polyketides | communols A, F and G are moderately active against *E. coli* and *Enterobacter aerogenes* | [37] |
| ***Penicillium oxalicum*** Ascomycota Eurotiomycetes | *Muricella flexuosa* Acanthagorgiidae | **oxalicumone A & B**  **(21)**  chromones | anticancer activity against A375 and SW-620 cancer cell lines; oxalicumone A is more potent | [38] |
| ***Penicillium* sp.** Ascomycota Eurotiomycetes | *Dichotella gemmacea* Ellisellidae | **6,8,50,60-tetrahydroxy-30-methylflavone**  polyketide | significant antifouling activity against *B. amphitrite* larvae settlement | [39] |
| ***Pestalotiopsis* sp.** Ascomycota Sordariomycetes | *Sarcophyton sp.* Alcyoniidae | **(+)- and ()-pestaloxazine A (16)**  polyketide-cyclopeptide | antiviral activity against EV71; ((+)-pestaloxazine A more potent and more selective | [40] |
| ***Pestalotiopsis* sp.** Ascomycota Sordariomycetes | *Sarcophyton sp.* Alcyoniidae | **()-pestalachloride D**  **(10)**  benzophenone derivative | moderate antibacterial activity against several Gram-negative bacteria | [41] |
| ***Phoma* sp.** Ascomycota Dothideomycetes | *Dichotella gemmacea* Ellisellidae | **phomaether A & C** dephenyl ethers | strong antibacterial activity against several Gram-positive and -negative pathogens | [42] |
| ***Scopulariopsis* sp.** *Ascomycota Sordariomycetes* | *Carijoa* sp*.*  Clavulariidae | **aniduquinolone A**  alkaloid | antifouling activity towards *B. amphitrite* larvae settlement | [43] |
| ***Scopulariopsis* sp.** Ascomycota Sordariomycetes | *Carijoa* sp.  Clavulariidae | **aflaquinolone A, D, F, G; 6-deoxyaflaquinolone E** alkaloid | antifouling activity towards *B. amphitrite* larvae settlement; deoxyaflaquinolone E shows broad spectrum antibacterial activity | [43] |
| ***Trichoderma aureoviride*** Ascomycota Sordariomycetes | *Annella sp.* Supergorgiidae | **trichodermaquinone** antthraquinone | weak activity against MRSA | [44] |
| ***Xylariaceae*** Ascomycota Sordariomycetes | *Melitodes squamata* Melithaeidae | **dicitrinin** (also penicitrinone) **A**  polyketide | strong antifouling activity against *B. neritina* larvae settlement | [45] |
| ***Xylariaceae*** Ascomycota Sordariomycetes | *Melitodes squamata* Melithaeidae | **(3R,4S)-(+)-4-hydroxy-6-deoxyscytalone**  polyketide | inhibitory activities towards SHP2, PTPlB, and IMPDH (anticancer) | [45] |
| ***Xylariaceae*** Ascomycota Sordariomycetes | *Melitodes squamata* Melithaeidae | **dihydrocitrinon**  polyketide | antifouling activity against *B. neritina* larvae settlement, inhibition of cathepsin B and the enzymes SHP2 and IMPDH (anticancer) | [45] |
| ***Xylariaceae*** Ascomycota Sordariomycetes | *Melitodes squamata* Melithaeidae | **phenol acid A**  polyketide | antifouling activity against *B. neritina* larvae settlement and cathepsin B inhibition (anticancer) | [45] |
| *Microalgae* |  |  |  |  |
| ***Symbiodinium* sp.**  Dinoflagellata  Dinophyceae | *Antillogorgia elisabethae*  Gorgoniidae | **Pseudopterosin A, B, C, D**  diterpene-glycoside | strong anti-inflammatory and analgesic activity | [46, 47] |

**MRSA** - methicillin resistant *Staphylococcus aureus.* **GABA** - *gamma-*aminobutyric acid neurotransmitter.

**Viruses:** **RSV** - respiratory syncytial virus; **PRRSV**- porcine reproductive and respiratory syndrome virus; **H1N1** and **H3N2**– Influenza A virus subtypes; **HSV-1** - Herpes simplex virus; **EV71** – human Enterovirus 71.

**Cancer cell lines:** **A375** – human malignant melanoma; **A549** – human lung carcinoma; **ACHN** - human kidney adenocarcinoma;

**Calu** – human lung carcinoma; **CNE-1** and **CNE-2** – nasopharyngeal carcinoma; **H460** – human lung cancer; **HCT-116** - human colon carcinoma; **HL-60** – human promyelocytic leukemia; **LoVo** – human colon metastasis; **MCF-7** - human breast cancer; **MOLT-4** – lymphoblastic leukemia; **Panc1** – human pancreas; **PC-3** - human prostate adenocarcinoma; **SW-620** – human colon adenocarcinoma; **T-47D** – human breast tumor.

**IMPDH** – Inosine-5'-monophosphate dehydrogenase; **SHP2** and **PTPlB** - protein tyrosine phosphatases; IMPDH, SHP2 & PTPlB are associated with cell proliferation; cathepsin B is upregulated in several cancers.

**(23*) versicolactone B** refers to compound 23 with the sum formula C_24_H_24_O_6_ as shown in figure 4 and described by [26,48]. However, the compound name likely needs revision since several *Aspergillus* derived lactones with different chemical structures and sum formula share this name.

**References**

1. Gao, C.-H.; Tian, X.-P.; Qi, S.-H.; Luo, X.-M.; Wang, P.; Zhang, S. Antibacterial and antilarval compounds from marine gorgonian-associated bacterium *Bacillus amyloliquefaciens* SCSIO 00856. *The Journal of Antibiotics* **2010**, *63*, 191–193, doi:10.1038/ja.2010.7.

2. Moree, W.J.; McConnell, O.J.; Nguyen, D.D.; Sanchez, L.M.; Yang, Y.-L.; Zhao, X.; Liu, W.-T.; Boudreau, P.D.; Srinivasan, J.; Atencio, L., et al. Microbiota of healthy corals are active against fungi in a light-dependent manner. *ACS Chemical Biology* **2014**, *9*, 2300–2308, doi:http://doi.org/10.1021/cb500432j.

3. Chen, Y.-H.; Lu, M.-C.; Chang, Y.-C.; Hwang, T.-L.; Wang, W.H.; Weng, C.F.; Kuo, J.; Sung, P.J. Pseudoalteromone A: a novel bioactive ubiquinone from a marine bacterium *Pseudoalteromonas* sp. CGH2XX (*Pseudoalteromonadaceae*) *Tetrahedron Letters* **2012**, *53*, 1675–1677, doi:10.1016/j.tetlet.2012.01.104.

4. Chen, Y.H.; Kuo, J.; Su, J.-H.; Hwang, T.L.; Chen, Y.H.; Lee, C.H.; Weng, C.F.; Sung, P.J. Pseudoalteromone B: A novel 15C compound from a narine bacterium *Pseudoalteromonas* sp. CGH2XX *Marine Drugs* **2012**, *10*, 1566-1571, doi:10.3390/md10071566

5. Fu, P.; Kong, F.; Wang, Y.; Wang, Y.; Liu, P.; Zuo, G.; Zhu, W. Antibiotic metabolites from the coral‐associated actinomycete *Streptomyces* sp. OUCMDZ‐1703. *Chinese Journal of Chemistry* **2013**, *31*, 100-104, doi:https://doi.org/10.1002/cjoc.201201062.

6. Al-Zereini, W.; Yao, B.C.F.F.; Laatsch, H.; Anke, H. Aqabamycins A-G: novel nitro maleimides from a marine *Vibrio* species: I. Taxonomy, fermentation, isolation and biological activities. *The Journal of Antibiotics* **2010**, *63*, 297–301, doi:10.1038/ja.2010.34.

7. Zheng, C.-J.; Shao, C.-L.; Guo, Z.-Y.; Chen, J.-F.; Deng, D.-S.; Yang, K.-L.; Chen, Y.-Y.; Fu, X.-M.; She, Z.-G.; Lin, Y.-C., et al. Bioactive hydroanthraquinones and anthraquinone dimers from a soft coral-derived *Alternaria* sp. fungus. *Journal of Natural Products* **2012**, *75*, 189-197, doi:10.1021/np200766d.

8. Zheng, C.J.; Shao, C.L.; Wu, L.Y.; Chen, M.; Wang, K.L.; Zhao, D.L.; Sun, X.P.; Chen, G.Y.; Wang , C.Y. Bioactive phenylalanine derivatives and cytochalasins from the soft coral-derived fungus, *Aspergillus elegans*. *Marine Drugs* **2013**, *10.3390/md11062054.*, 2054-2068, doi:10.3390/md11062054.

9. Afiyatullov, S.S.; Zhuravleva, O.I.; Chaikina, E.L.; Anisimov, M.M. A new spirotryprostatin from the marine isolate of the fungus *Aspergillus fumigatus*. *Chemistry of Natural Compounds* **2012**, *48*.

10. Peng, X.; Wang, Y.; Zhu, T.; Zhu, W. Pyrazinone derivatives from the coral-derived *Aspergillus ochraceus* LCJ11-102 under high iodide salt. *Archives of Pharmacal Research* **2018**, *41*, 184–191.

11. Chen, M.; Shao, C.L.; Fu, X.M.; Kong, C.J.; She, Z.G.; Wang, C.Y. Lumazine peptides penilumamides B-D and the cyclic pentapeptide asperpeptide A from a gorgonian-derived *Aspergillus sp. fungus*. *Journal of Natural Products* **2014**, *77*, 1601-1606, doi:10.1021/np5001686.

12. Chen, M.; Shao, C.L.; Meng, H.; She, Z.G.; Wang, C.Y. Anti-respiratory syncytial virus prenylated dihydroquinolone derivatives from the gorgonian-derived fungus *Aspergillus* sp. XS-20090B15. *Journal of Natural Products* **2014**, *77*, 2720-2724, doi:10.1021/np500650t.

13. Bao, J.; Zhang, X.-Y.; Xu, X.-Y.; He, F.; Nong, X.-H.; Qi, S.-H. New cyclic tetrapeptides and asteltoxins from gorgonian-derived fungus *Aspergillus* sp. SCSGAF 0076. *Tetrahedron* **2013**, *69*, 2113-2117.

14. Shao, C.-L.; Wanga, C.-Y.; Wei, M.-Y.; Gu, Y.-C.; She, Z.-G.; Qian, P.-Y.; Lin, Y.-C. Aspergilones A and B, two benzylazaphilones with an unprecedented carbon skeleton from the gorgonian-derived fungus *Aspergillus* sp. *Bioorganic and Medicinal Chemistry Letters* **2011**, *21*, 690–693, doi: 10.1016/j.bmcl.2010.12.005.

15. Chen, M.; Shao, C.L.; Kong, C.J.; She, Z.C.; Wang, C.Y. A new anthraquinone derivative from a gorgonian-derived fungus *Aspergillus* sp. *Chemistry of Natural Compounds* **2014**, *50*, 617-620, doi:0009-3130/14/5004-0617.

16. Bao, J.; Xu, X.-Y.; Zhang, X.-Y.; Qi, S.-H. A new macrolide from a marine-derived fungus *Aspergillus* sp. *Natural Product Communications* **2013**, *8*, 1127 - 1128.

17. Xu, X.; He, F.; Zhang, X.; Bao, J.; Qi, S. New mycotoxins from marine-derived fungus *Aspergillus* sp. SCSGAF0093. *Food and Chemical Toxicology* **2013**, *53*, 46-51, doi:10.1016/j.fct.2012.11.037.

18. Xu, X.Y.; Zhang, X.Y.; He, F.; Peng, J.; Nong, X.H.; Qi, S.-H. Two new compounds from gorgonian-associated fungus *Aspergillu*s sp. . *Natural Product Communications* **2013**, *8*, 1069-1070

19. Wei, M.-Y.; Chen, G.-Y.; Wang, Y.; Zhang, X.-L.; Wang, C.-Y.; Shao, C.-L. Isolation, 1H, 13C NMR assignments, and crystal structure of chrodrimanin B from a marine fungus *Aspergillus sp.* *Chemistry of Natural Compounds* **2011**, *47*, 571-573.

20. Xu, Y.; Furutani, S.; Ihara, M.; Ling, Y.; Yang, X.; Kai, K.; Hayashi, H.; Matsuda, K. Meroterpenoid chrodrimanins are selective and potent blockers of insect GABA-gated chloride channels. *Plos One* **2015**, *10*, e0122629, doi:10.1371/journal.pone.0122629.

21. Wei, M.Y.; Wang, C.Y.; Liu, Q.A.; Shao, C.L.; She, Z.G.; Lin, Y.C. Five sesquiterpenoids from a marine-derived fungus *Aspergillus sp.* isolated from a gorgonian *Dichotella gemmacea*. *Marine Drugs* **2010**, *8*, 941-949, doi:10.3390/md8040941.

22. He, F.; Han, Z.; Peng, J.; Qian, P.-Y.; Qi, S.-H. Antifouling indole alkaloids from two marine derived fungi. *Natural Product Communications* **2013**, *8*, 329 - 332.

23. Parvatkar, R.R.; D’Souza, C.; Tripathi, A.; Naik, C.G. Aspernolides A and B, butenolides from a marine-derived fungus *Aspergillus terreus*. *Phytochemistry* **2009**, *70*, 128–132.

24. He, F.; Bao, J.; Zhang, X.-Y.; Tu, Z.-C.; Shi, Y.-M.; Qi, S.-H. Asperterrestide A, a cytotoxic cyclic tetrapeptide from the marine-derived fungus *Aspergillus terreus* SCSGAF0162. *Journal of Natural Products* **2013**, *76*, 1182–1186, doi:10.1021/np300897v.

25. Nong, X.H.; Wang, Y.F.; Zhang, X.Y.; Zhou, M.P.; Xu, X.Y.; Qi, S.H. Territrem and butyrolactone derivatives from a marine-derived fungus *Aspergillus terreus*. *Marine Drugs* **2014**, *12*, 6113-6124, doi:doi: 10.3390/md12126113.

26. Liu, M.; Zhou, Q.; Wang, J.; Liu, J.; Qi, C.; Lai, Y.; Zhu, H.; Xue, Y.; Hu, Z.; Zhang, Y. Anti-inflammatory butenolide derivatives from the coral-derived fungus *Aspergillus terreus* and structure revisions of aspernolides D and G, butyrolactone VI and 4′,8′′-diacetoxy butyrolactone VI. *RSC Advances* **2018**, *8*, 13040-13047, doi:10.1039/C8RA01840E.

27. Chen, M.; Fu, X.M.; Kong, C.J.; Wang, C.Y. Nucleoside derivatives from the marine-derived fungus *Aspergillus versicolor*. *Natural Product Research* **2014**, *28*, 895-900, doi:10.1080/14786419.2014.891114.

28. Zhuang, Y.; Teng, X.; Wang, Y.; Liu, P.; Wang, H.; Li, J.; Li, G.; Zhu , W. Cyclopeptides and polyketides from coral-associated fungus, *Aspergillus versicolor* LCJ-5-4. *Tetrahedron* **2011**, *67*, 7085-7089.

29. Zhuang, Y.; Teng, X.; Wang, Y.; Liu, P.; Li, G.; Zhu, W. New quinazolinone alkaloids within rare amino acid residue from coral-associated fungus, *Aspergillus versicolor* LCJ-5-4. *Organic Letters* **2011**, *13*, 1130–1133, doi:10.1021/ol103164n.

30. Li, H.-J.; Jiang, W.-H.; Liang, W.-L.; Huang, J.-X.; Mo, Y.-F.; Ding, Y.-Q.; Lam, C.-K.; Qian, X.-J.; Zhu, X.-Z.; Lan, W.-J. Induced marine fungus *Chondrostereum* sp. as a means of producing new sesquiterpenoids chondrosterins I and J by using glycerol as the carbon source. *Marine Drugs* **2014**, *12*, 167–175, doi:http://doi.org/10.3390/md12010167.

31. Li, H.-J.; Xie, Y.-L.; Xie, Z.-L.; Chen, Y.; Lam, C.-K.; Lan, W.-J. Chondrosterins A–E, triquinane-type sesquiterpenoids from soft coral-associated fungus *Chondrostereum* sp. *Marine Drugs* **2012**, *10*, 627-638, doi:doi:10.3390/md10030627.

32. Li, H.-J.; Chen, T.; Xie, Y.-L.; Chen, W.-D.; Zhu, X.-F.; Lan, W.-J. Isolation and structural elucidation of chondrosterins F–H from the marine fungus *Chondrostereum* sp. *Marine Drugs* **2013**, *11*, 551-558, doi:doi:10.3390/md11020551.

33. Li, H.-J.; Lan, W.-J.; Lam, C.-K.; Yang, F.; Zhu, X.-F. Hirsutane sesquiterpenoids from the marine-derived fungus *Chondrostereum* sp. *Chemistry and Biodiversity* **2011**, *8*, 317-324, doi:10.1002/cbdv.201000036.

34. Shao, C.L.; Wu, H.X.; Wang, C.Y.; Liu, Q.A.; Xu, Y.; Wei, M.Y.; Qian, P.Y.; Gu, Y.C.; Zheng, C.J.; She, Z.G., et al. Potent antifouling resorcylic acid lactones from the gorgonian-derived fungus *Cochliobolus lunatus*. *Journal of Natural Products* **2011**, *74*, 629-633, doi:10.1021/np100641b.

35. Kasettrathat, C.; Ngamrojanavanich, N.; Wiyakrutta, S.; Mahidol, C.; Ruchirawat, S.; Kittakoop, P. Cytotoxic and antiplasmodial substances from marine-derived fungi, *Nodulisporium* sp. and CRI247-01. *Phytochemistry* **2008**, *69*, 2621–2626.

36. Khamthong, N.; Rukachaisirikul, V.; Phongpaichit, S.; Preedanon, S.; Sakayaroj, J. Bioactive polyketides from the sea fan-derived fungus *Penicillium citrinum* PSU-F51. *Tetrahedron* **2010**, *68*, 8245-8250.

37. Wang, J.; Liu, P.; Wang, Y.; Wang, H.; Li, J.; Zhuang, Y.; Zhu, W. Antimicrobial aromatic polyketides from gorgonian‐associated fungus, *Penicillium commune* 518 *Chinese Journal of Chemistry* **2012**, *30*, 1236-1242, doi:https://doi.org/10.1002/cjoc.201100640.

38. Sun, Y.-L.; Bao, J.; Liu, K.-S.; Zhang, X.-Y.; He, F.; Wang, Y.F.; Nong, X.-H.; Qi, S.-H. Cytotoxic dihydrothiophene-condensed chromones from the marine-derived fungus *Penicillium oxalicum*. *Planta Medica* **2013**, *79*, 1474-1479, doi:10.1055/s-0033-1350805.

39. Bao, J.; Sun, Y.L.; Zhang, X.Y.; Han, Z.; Gao, H.C.; He, F.; Qian, P.Y.; Qi, S.H. Antifouling and antibacterial polyketides from marine gorgonian coral-associated fungus *Penicillium* sp. SCSGAF 0023. *The Journal of Antibiotics* **2013**, *66*, 219–223 doi:10.1038/ja.2012.110.

40. Jia, Y.-L.; Wei, M.-Y.; Chen, H.-Y.; Guan, F.-F.; Wang, C.-Y.; Shao, C.-L. (+)- and (−)-Pestaloxazine A, a pair of antiviral enantiomeric alkaloid dimers with a symmetric spiro [oxazinane-piperazinedione] skeleton from *Pestalotiopsis* sp. *Organic Letters* **2015**, *17*, 4216-4219, doi:10.1021/acs.orglett.5b01995.

41. Wei, M.Y.; Li, D.; Shao, C.L.; Deng, D.S.; Wang, C.Y. (±)-Pestalachloride D, an antibacterial racemate of chlorinated benzophenone derivative from a soft coral-derived fungus *Pestalotiopsis sp.* *Marine Drugs* **2013**, *11*, 1050-1060, doi:10.3390/md11041050.

42. Shi, T.; Qi, J.; Shao, C.L.; Zhao , D.L.; Hou , X.M.; C.Y., W. Bioactive diphenyl ethers and isocoumarin derivatives from a gorgonian-derived fungus *Phoma* sp. (TA07-1). *Marine Drugs* **2017**, *15*, doi:10.3390/md15060146

43. Shao, C.L.; Xu, R.F.; Wang, C.Y.; Qian, P.Y.; Wang, K.L.; Wei, M.Y. Potent antifouling marine dihydroquinolin-2(1H) -one-containing alkaloids from the gorgonian coral-derived fungus *Scopulariopsis* sp. *Marine Biotechnology* **2015**, *17*, 408–415 doi:10.1007/s10126-015-9628-x.

44. Khamthong, N.; Rukachaisirikul, V.; Tadpetch, K.; Kaewpet, M.; Phongpaichit, S.; Preedanon, S.; Sakayaroj, J. Tetrahydroanthraquinone and xanthone derivatives from the marine-derived fungus *Trichoderma aureoviride* PSU-F95. *Archives of Pharmacal Research* **2012** *35*, 461-468, doi:10.1007/s12272-012-0309-2.

45. Nong, X.H.; Zheng, Z.H.; Zhang, X.-Y.; Lu, X.H.; Qi, S.H. Polyketides from a marine-derived fungus *Xylariaceae* sp. . *Marine Drugs* **2013**, *11*, 1718-1727, doi:10.3390/md11051718

46. Look, S.A.; Fenical, W.; Robert, S.J.; Clardy, J. The Pseudopterosins: Anti-inflammatory and analgesic natural products from the sea whip *Pseudopterogorgia elisabethae*. *Proceedings of the National Academy of Sciences of the United States of America* **1986**, *83*, 6238-6240.

47. Mydlarz, L. D.; Jacobs, R.S.; Boehnlein, J.; Kerr, R.G. "Pseudopterosin biosynthesis in *Symbiodinium* sp., the dinoflagellate symbiont of *Pseudopterogorgia elisabethae*." *Chemistry & Biology* **2003,** 10, 1051–1056.

48. Zhou, M.; Du, G.; Yang, H.Y.; Xia, C.F.; Yang, J.X.; Ye, Y.; Gao, X.M.; Li, X.N.; Hu, Q.F. Antiviral butyrolactones from the endophytic fungus *Aspergillus versicolor*. *Planta Medica* **2015**, *8*, 235-240, doi:10.1055/s-0034-1396153.
