# Supplementary material for: Bioactive Secondary Metabolites from Octocoral-Associated Microbes—New Chances for Blue Growth
Source: Mar Drugs. 2018 Dec 4;16(12):485. doi: 10.3390/md16120485 (PMC6316421; doi:10.3390/md16120485)
Supplement: Supplementary file 1 [file marinedrugs-16-00485-s001.zip › Supplementary_revised/Table S2.docx]

| **Soft coral host** | **Bacterial class** | **Bacterial strain** | **Alginate lyases** | **Amylases** | **Cellulases** | **Chitinases** | **Proteases** |
| --- | --- | --- | --- | --- | --- | --- | --- |
| ***Eunicella labiata*** | *α-proteo-bacteria* | *Ruegeria* sp. EL01 | 0 | 1 | 0 | 0 | 16 |
|  |  | *Roseovarius* sp. EL26 | 0 | 0 | 0 | 0 | 14 |
|  |  | *Pseudophaeobacter* sp. EL27 | 0 | 0 | 0 | 1 | 14 |
|  |  | *Sulfitobacter* sp. EL44 | 0 | 0 | 0 | 0 | 14 |
|  |  | uncl. *Rhodobacteraceae* EL53 | 0 | 0 | 0 | 0 | 14 |
|  |  | uncl. *Rhodobacteraceae* EL129 | 0 | 0 | 0 | 0 | 12 |
|  |  | *Labrenzia* sp. EL143 | 0 | 5 | 2 | 0 | 18 |
|  |  | *Sphingorhabdus* sp. EL138 | 0 | 0 | 0 | 0 | 20 |
|  |  | *Kiloniella* sp. EL199 | 0 | 0 | 0 | 0 | 14 |
|  | *γ-proteo-bacteria* | *Aliivibrio* sp. EL58 | 0 | 8 | 0 | 5 | 19 |
|  | *flavo-bacteria* | *Aquimarina* sp. EL33 | 0 | 9 | 4 | 2 | 36 |
| ***Eunicella verrucosa*** | *γ-proteo-bacteria* | *Vibrio* sp. Evd3 | 8 | 24 | 1 | 5 | 13 |
|  |  | *Vibrio* sp. Evd11 | 1 | 20 | 1 | 5 | 17 |
|  |  | *Vibrio* sp. Evh12 | 8 | 16 | 1 | 4 | 15 |
|  |  | *Vibrio* sp. Evh13 | 0 | 22 | 1 | 5 | 15 |

**Table S2.** Copy number of open reading frames (ORFs) of biocatalyst protein families (PFAM) present on

the genomes of 15 octocoral-associated bacteria.

For each (amino-acid translated) bacterial genome, PFAMs were predicted using the WebMGA platform (<http://weizhong-lab.ucsd.edu/metagenomic-analysis/>), and PFAM entries relevant for amylase (*n* = 3), alginate lyase (*n* = 1), cellulase (*n* = 2), chitinase (*n* = 3), and protease (*n* = 17) were selected. The table shows the sum of ORFs for all PFAM entries for each enzyme. PFAMS related to the respective binding proteins of the substrate are not included. PFAM entries included: amylase (PF00128.17, PF02806.11, PF02903.7), alginate lyase (PF08787.4), cellulase (PF00150.11, PF02927.7), chitinase (PF06483.4, PF00182.12, PF08329.3), and protease (PF00026.16, PF00077.13, PF00112.16, PF00574.16, PF00814.18, PF00949.14, PF01421.12, PF02073.8, PF02190.9, PF02517.9, PF05362.6, PF09668.3, PF10026.2, PF11818.1, PF04586.10).
